# Barrier-to-autointegration factor 1 (Banf1) regulates poly [ADP-ribose] polymerase 1 (PARP1) activity following oxidative DNA damage

**DOI:** 10.1038/s41467-019-13167-5

**Published:** 2019-12-03

**Authors:** Emma Bolderson, Joshua T. Burgess, Jun Li, Neha S. Gandhi, Didier Boucher, Laura V. Croft, Samuel Beard, Jennifer J. Plowman, Amila Suraweera, Mark N. Adams, Ali Naqi, Shu-Dong Zhang, David A. Sinclair, Kenneth J. O’Byrne, Derek J. Richard

**Affiliations:** 1 Cancer & Ageing Research Program, Institute of Health and Biomedical Innovation at the Translational Research Institute (TRI), Queensland University of Technology (QUT), Brisbane, Queensland Australia; 2 Princess Alexandra Hospital, Ipswich Road, Woolloongabba, Brisbane, Queensland 4102 Australia; 3 Department of Genetics, Paul F. Glenn Center for Biology of Aging Research, Harvard Medical School, Boston, MA 02115 USA; 4 National Key Laboratory of Medical Molecular Biology, Department of Biochemistry and Molecular Biology, Institute of Basic Medical Sciences, Chinese Academy of Medical Sciences, Peking Union Medical College, Beijing, 100005 China; 5 School of Mathematical Sciences, Queensland University of Technology, Brisbane, 4000 Queensland Australia; 6 Department of Chemistry, Pennsylvania State University, University Park, PA USA; 7 Northern Ireland Centre for Stratified Medicine, University of Ulster, Londonderry, UK; 8 The Department of Pharmacology, School of Medical Sciences, The University of New South Wales, Sydney, New South Wales 2052 Australia

**Keywords:** Cell biology, Single-strand DNA breaks

## Abstract

The DNA repair capacity of human cells declines with age, in a process that is not clearly understood. Mutation of the nuclear envelope protein barrier-to-autointegration factor 1 (Banf1) has previously been shown to cause a human progeroid disorder, Néstor–Guillermo progeria syndrome (NGPS). The underlying links between Banf1, DNA repair and the ageing process are unknown. Here, we report that Banf1 controls the DNA damage response to oxidative stress via regulation of poly [ADP-ribose] polymerase 1 (PARP1). Specifically, oxidative lesions promote direct binding of Banf1 to PARP1, a critical NAD^+^-dependent DNA repair protein, leading to inhibition of PARP1 auto-ADP-ribosylation and defective repair of oxidative lesions, in cells with increased Banf1. Consistent with this, cells from patients with NGPS have defective PARP1 activity and impaired repair of oxidative lesions. These data support a model whereby Banf1 is crucial to reset oxidative-stress-induced PARP1 activity. Together, these data offer insight into Banf1-regulated, PARP1-directed repair of oxidative lesions.

## Abstract (web summary)

Mutation of the nuclear envelope protein, barrier-to-autointegration factor 1 (Banf1), has previously been associated with the development of ageing associated diseases in a human progeria syndrome. Here, the authors reveal the functional link between Banf1-regulated, PARP1-directed repair of oxidative lesions.

## Introduction

During the ageing process DNA repair mechanisms deteriorate, leading to the accumulation of DNA damage, contributing toward the development of ageing-associated diseases, such as cancer, osteoporosis and Alzheimer’s disease^1–4^. Barrier-to-autointegration factor 1 (Banf1) is a DNA-binding protein that functions to tether DNA to structural proteins located on the nuclear envelope^5^. Strikingly, a point mutation in *Banf1*, alanine 12 to threonine (A12T), is associated with a severe premature ageing syndrome, Néstor–Guillermo progeria syndrome (NGPS), characterised by pathologies usually associated with ageing, such as generalised lipoatrophy and severe osteoporosis^6,7^. Premature ageing is intrinsically linked with genome stability. For example, the genes mutated in other well-characterised progeria syndromes such as Werner Syndrome and Hutchinson–Gilford (Progeria) Syndrome result from mutations in *WRN* and *Lamin A* genes, respectively and are implicated in DNA repair and genome stability^1,8^.

The poly [ADP-ribose] (PAR) polymerase 1 (PARP1) protein responds rapidly to DNA strand breaks and oxidative DNA damage, using NAD^+^ to catalyse auto-ADP-ribosylation, adding long, branched PAR chains up to 200 residues in size onto serine and glutamic residues in the PARP1 automodification domain^9–14^. These serve to further activate PARP1, promoting the recruitment of other DNA repair proteins involved in the repair process, including XRCC1 (X-ray repair cross-complementing protein 1), and DNA end-processing kinase/phosphatase PNK (bifunctional polynucleotide phosphatase/kinase)^15^. Many PARP1 substrates have been identified, including targets with roles in DNA repair, transcription and regulation of chromatin structure. Recent studies have identified that in addition to glutamic residues, PARP1 substrates may also be ADP-ribosylated on serine or tyrosine residues^9–14,16^. The catalytic domain of PARP1 is responsible for three enzymatic reactions during synthesis of the PAR chains, initiation, elongation and branching.

Increased PARP1 activity has been shown to be associated with improved health and longevity^17–19^. Thus, increasing our understanding of PARP1 regulation is of critical importance and has implications for ageing-associated diseases such as cancer^20,21^.

We present here evidence that Banf1 functions in DNA repair and genome stability pathways through the direct regulation of PARP1 poly-ADP-ribose polymerase activity. Specifically, Banf1 relocalises from the nuclear envelope following oxidative stress and binds directly to PARP1 to inhibit auto-poly-ADP-ribose activity. In addition, we also show that mutation of Banf1 in a human progeria syndrome impacts upon PARP1 activity and subsequent DNA repair.

## Results

### Banf1 responds to oxidative stress

**Fig. 1:**
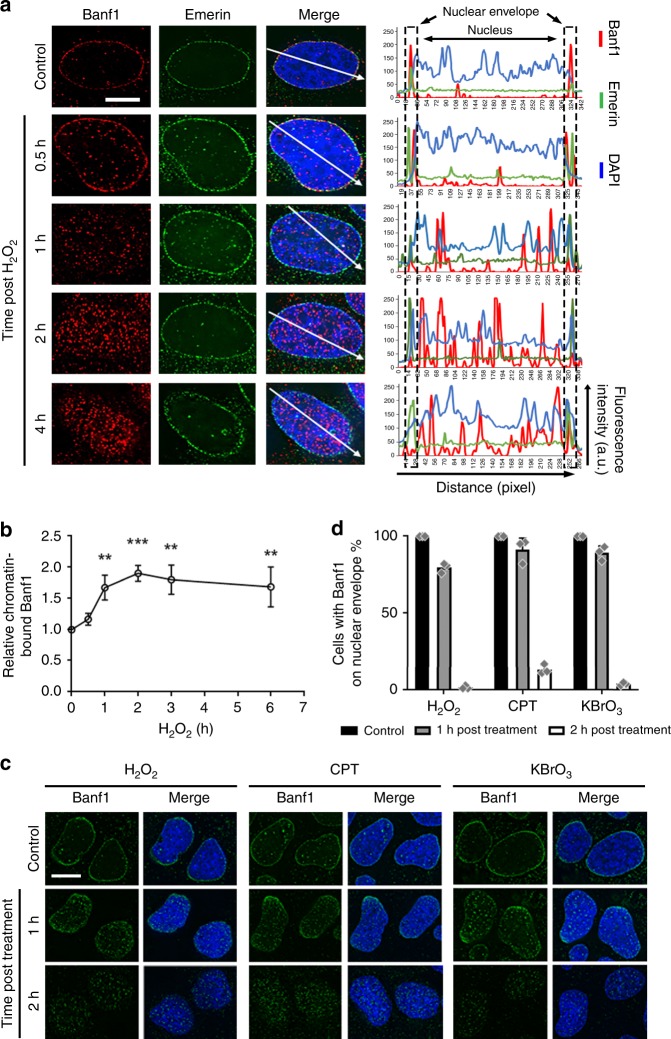
Banf1 responds to oxidative stress. **a** Banf1 relocalises from the nuclear envelope following oxidative stress induced by 200 μM H_2_O_2_ in U2OS cells. Representative cells are shown. **b** The nuclear intensity of Banf1 in U2OS cells treated as in (**a**), were analysed via an InCell Analyser 2200. One-way ANOVA was used for statistical analysis. **c** Banf1 relocalisation following hydrogen peroxide (H_2_O_2_), camptothecin (CPT), or potassium bromate (KBrO_3_) at the indicated times post compound removal. Representative cells stained with the indicated antibodies are shown. **d** Nuclear envelope localisation (from (**c**)) was assessed using a delta vision PDV microscope. Immunofluorescence scale bars represents 10 μm. Histogram data shown represent the mean and S.D. of three independent experiments. ANOVA **P < 0.01, ***P < 0.001. Source data are provided as a Source Data file.

One of the main characteristics of proteins that are mutated in premature ageing syndromes is that they are involved in the repair of DNA damage^8^. Given that mutation of Banf1 leads to a premature ageing syndrome, we reasoned that Banf1 may also play a role in the repair of DNA damage. In unperturbed cells, Banf1 can be detected in pre-extracted cells, to be localised to the nuclear envelope^5^. However, following induction of oxidative stress by H_2_O_2_, that primarily induces oxidised DNA bases in the form of 8-Oxo-Guanine (8-OxoG) lesions^22^, Banf1 relocalised from the nuclear envelope to the chromatin between 1- and 2-h post H_2_O_2_ removal (Fig. 1a, b). This was not due to nuclear envelope breakdown as the Banf1-interacting protein Emerin (EMD) remained on the nuclear envelope following H_2_O_2_ treatment (Fig. 1a). This response to H_2_O_2_ was compared to another oxidising agent, that also primarily induces 8-OxoG lesions^23^, potassium bromate (KBrO_3_) and the topoisomerase I inhibitor, camptothecin (CPT). Banf1 was observed to respond similarly to H_2_O_2_, KBrO_3_ and CPT and could not be detected on the nuclear envelope within 2 h of treatment (Fig. 1c, d). CPT initially induces single-strand DNA breaks that are processed into double-strand breaks during the S-phase of the cell cycle^24^. Notably, Banf1 relocalised from the nuclear envelope within 2 h of camptothecin treatment in the majority of cells, indicating this was not solely an S-phase or DNA double-strand break response (as marked by γ-H2AX), suggesting that in contrast to γ-H2AX Banf1 may respond to DNA single-strand breaks, before they are converted to double-strand breaks in S-phase (Supplementary Fig. 1a, b). Images of cells fixed without prior treatment with extraction buffer have been included as a comparison for Banf1 localisation in soluble fractions and illustrates that relocalisation of Banf1 can not be detected in cells that have not been treated with extraction buffer (Supplementary Fig. 1c). Banf1 can be detected in both the cytoplasmic and chromatin-bound fractions in unperturbed cells and H_2_O_2_ treatment induces increases in Banf1 protein levels in the chromatin fraction and total cell lysates following H_2_O_2_ treatment (Supplementary Fig. 1d, e).

### Banf1 interacts with PARP1

**Fig. 2:**
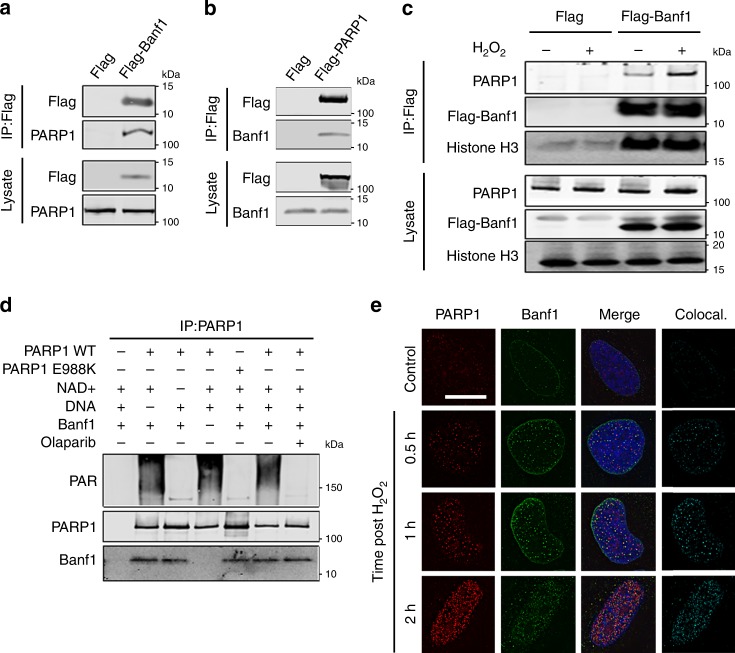
Banf1 binds to PARP1 following oxidative stress. **a**, **b** Banf1 and PARP1 are in a complex; immunoprecipitations from HEK293T cells expressing Flag, Flag-Banf1 or Flag-PARP1 using Flag antibodies. Immunoprecipitates were immunoblotted with the indicated antibodies. **c** The Banf1:PARP1 interaction after H_2_O_2_; immunoprecipitations from HEK293T cells ectopically expressing Flag or Flag-Banf1 1 h after H_2_O_2_ removal. Immunoprecipitates were immunoblotted with the indicated antibodies. **d** Banf1 and PARP1 directly interact. The indicated purified proteins (±NAD^+^/DNA/olaparib) were incubated together before immunoprecipitation with PARP1 antibodies and immunoblotting with the indicated antibodies. **e** Banf1 and PARP colocalise following oxidative stress induced by H_2_O_2_ in U2OS cells. Representative cells fixed at the indicated times following removal of H_2_O_2_ and stained with the indicated antibodies are shown, the colocalisation was analysed using ImageJ. The scale bar represents 10 μm. Immunoblots are representative of three independent experiments. Source data are provided as a Source Data file.

Given that the Banf1 protein responded to oxidative stress and single-strand breaks, we next reasoned that Banf1 may regulate other proteins involved in these repair pathways. PARP1 is required for repair of DNA single-strand breaks and as such is involved in the repair of both oxidative lesions (in which single-strand breaks are produced as a by-product of base excision repair) and direct single-strand breaks^20^. PARP1 has previously been identified as a Banf1 interactor in a large-scale proteomics screen^25^. We demonstrated an interaction between Banf1 and PARP1 in human embryonic kidney (HEK) 293T cells expressing Flag-Banf1 or Flag-PARP1 (Fig. 2a, b) and also between endogenous proteins in 293T cells and human skin fibroblasts, confirming that Banf1 and PARP1 form a complex (Supplementary Fig. 2a, b). Furthermore, this interaction was increased by H_2_O_2_-induced oxidative stress (Fig. 2c, Supplementary Fig. 2b, c). The interaction was shown to be direct and remained intact in the absence of NAD^+^ and DNA, in the presence of PARP1 inhibitors and when PARP1 was catalytically inactive (PARP1 E988K) in vitro and in cells (Fig. 2d, Supplementary Fig. 2d–f), suggesting the binding is independent of Poly-ADP-ribose activity. Consistent with the interaction between Banf1 and PARP1 following oxidative DNA damage, we could also observe their colocalisation via immunofluorescence (Fig. 2e). The Pearson coefficient *r* values were 0.24, prior to H_2_O_2_ and 0.38 and 0.48, 0.5 h and 1 h following removal of H_2_O_2_, respectively.

### Banf1 regulates PARP1 poly-ADP-ribose activity

**Fig. 3:**
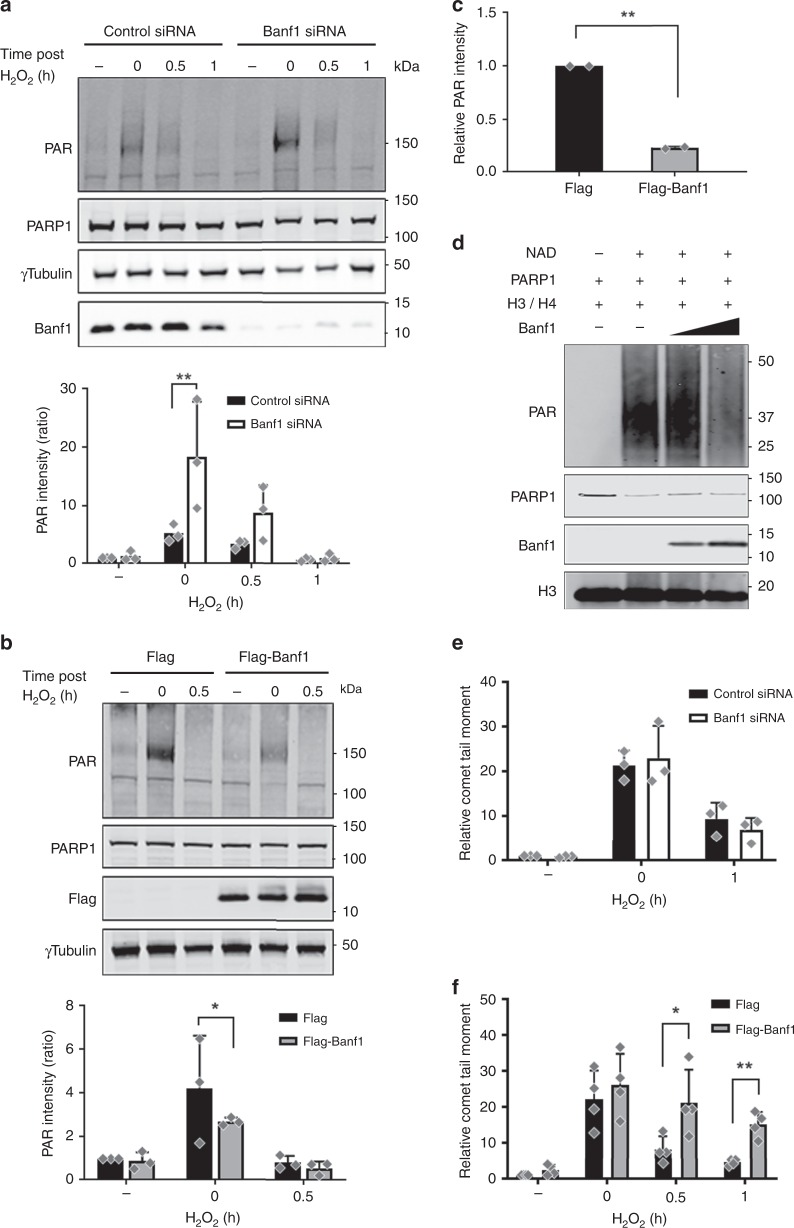
Banf1 regulates PARP1 activity and repair of oxidative DNA damage. **a** Auto-poly-ADP-ribosylation of PARP1 in U2OS cells depleted of Banf1 following H_2_O_2_. The PAR bands were analysed via densitometry and normalised to γ-Tubulin. Two-way ANOVA was used for statistical analysis. **b** Auto-poly-ADP-ribose of PARP1 in U2OS expressing ectopic Flag-Banf1 following H_2_O_2_. The PAR bands were analysed via densitometry and normalised to γ-Tubulin. Two-way ANOVA was used for statistical analysis. **c** Inhibition of poly-ADP-ribose activity of PARP1 purified from HEK293T cells expressing ectopic Flag-Banf1 on an immobilised histone substrate following H_2_O_2_. Data shown represent the mean and S.D. of two independent experiments. Paired t-test was used for statistical analysis. **d** In vitro inhibition of PARP1 poly-ADP-ribose activity on histones by purified Banf1. **e** Alkaline comet assay showing the relative olive tail moment in Banf1-deficient and control U2OS cells. **f** Banf1 inhibits repair of oxidative DNA damage. Alkaline comet assay showing the relative olive tail moment in U2OS control cells and cells ectopically expressing Flag-Banf1. Paired *t* test was used for statistical analysis. Histogram data shown in **f** represent the mean and S.D. of four independent experiments immunoblots are representative of three independent experiments. Unless otherwise stated, histogram data shown represent the mean and S.D. of three independent experiments. ANOVA was used for statistical analysis. *P < 0.05, **P < 0.01. Source data are provided as a Source Data file.

We next examined the effect of Banf1 on PARP1 activity. Cells depleted of Banf1 demonstrated increased PARP1 auto-ADP-ribosylation activity following H_2_O_2_ treatment (Fig. 3a, Supplementary Fig. 3a, b). Conversely, ectopic overexpression of Banf1, reproducibly decreased PARP1 auto-ADP-ribosylation, suggesting that Banf1 negatively regulates PARP1 activity (Fig. 3b). This effect was also observed in vitro (Supplementary Fig. 3c). Consistent with the defect in auto-ADP-ribosylation, PARP1 activity towards histones was significantly inhibited in the presence of Banf1 (Fig. 3c). Similarly, the direct ADP-ribosylation of histones H3/H4 was also significantly inhibited by Banf1 in a reconstituted assay (Fig. 3d). Following DNA damage poly-ADP-ribosylation is rapidly removed by the poly-ADP-glycohydrolase, PARG. Treatment of cells overexpressing Banf1 with a PARG inhibitor was unable to correct PARP1 ADP-ribosylation to the same extent as control cells, suggesting that the effect of Banf1 on PARP1 ADP-ribosylation was not due to upregulation of poly-ADP-ribose removal by PARG (Supplementary Fig. 3d).

Despite co-localising on the chromatin following H_2_O_2_, Banf1 and PARP1 recruitment to chromatin were mutually independent (Supplementary Fig. 4a, d) and both chromatin-bound Banf1 and PARP1 levels were similar in cells depleted of the other protein before and after H_2_O_2_ (Supplementary Fig. 4b, c).

### Banf1 regulates the repair of oxidative lesions

Depletion of Banf1 led to a small decrease in H_2_O_2_-induced comet tail length under alkaline conditions, but this did not reach statistical significance (Fig. 3e, Supplementary Fig. 4e). In contrast, overexpression of Banf1 resulted in an increase of H_2_O_2_-induced comet tail length, indicating that the repair of oxidised DNA lesions was impaired, consistent with the inhibition of PARP1 activity in cells over-expressing Banf1 (Fig. 3f, Supplementary Fig. 4f).

### Banf1 interacts with the NAD^+^-binding domain of PARP1

**Fig. 4:**
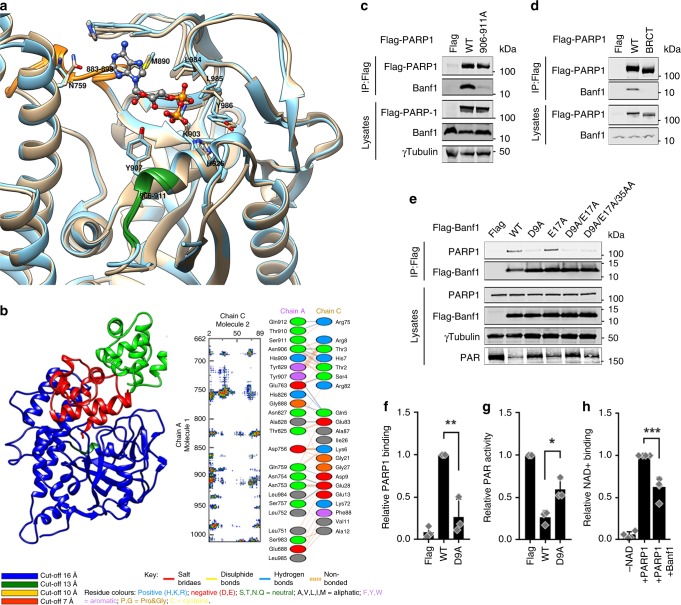
The N-terminal of Banf1 binds to the NAD-binding domain of PARP1. **a** Structural superimposition of catalytic domains of chicken (blue colour) and human (golden colour) PARP1. NAD^+^ analogue is shown as ball and sticks and the conserved binding site residues shown as sticks. The loop consisting of residues 906–911 and 883–893 of PARP1 are highlighted in dark green and orange, respectively. **b** 3D structure of the most representative catalytic domain of PARP1–Banf1 obtained from Cluspro docking server. The Banf1 dimer is shown in red and green ribbons. The N-terminal of Banf1 monomer occupies the NAD^+^ binding site hence inhibiting NAD^+^ interaction. The interface residues and interactions at the PARP1–Banf1 interface are shown in the box where chain A and C represents residues from PARP1 and Banf1, respectively. The loop consisting of residues 906–911 of PARP1 are highlighted in dark green. Single-letter abbreviations for the amino acid residues are as follows: A, Ala; E, Glu, D, Asp; N, Asn. **c**, **d** Interactions of Banf1 with Flag-PARP1 mutants. Flag immunoprecipitations from HEK293T cells ectopically expressing the indicated Flag-PARP1 proteins 1 h following H_2_O_2_ removal. **e** Interactions of PARP1 with Flag-Banf1 mutants. Flag immunoprecipitations from HEK293T cells ectopically expressing the indicated Flag-Banf1 proteins 1 h following H_2_O_2_ removal. **f** Analysis of the relative Banf1 WT vs. D9A binding to PARP1 via immunoprecipitation. **g** Analysis of the relative auto-poly-ADP-ribosylation of PARP1 in cells expressing WT or D9A Banf1. **h** In vitro inhibition of PARP1 binding NAD^+^ in the presence of purified Banf1. Histogram data shown in h, represent the mean and S.D. of four independent experiments. Immunoblots are representative of three independent experiments. Unless otherwise stated, histogram data shown represent the mean and S.D. of three independent experiments. Unpaired *t* test was used for statistical analysis. *P < 0.05, **P < 0.01, ***P < 0.001. Source data are provided as a Source Data file.

In order to shed light on the mechanism of Banf1-mediated regulation of PARP1 poly-ADP-ribosylation, we next characterised the PARP1–Banf1 interaction, by modelling of protein:protein interactions using molecular docking. This indicated that the N-terminus of the Banf1 monomer bound adjacent to the NAD^+^ binding site of PARP1 (Fig. 4a, b). The surface contacts of this PARP1–Banf1 complex were analysed further and the interaction surface showed good surface complementarity with numerous interacting residues (Fig. 4b). The PARP1–Banf1 complex was stable during the 100 ns molecular dynamics (MD) simulation. To predict the effect of Banf1 binding on PARP1 activity, we also superimposed Banf1 onto a model of full-length PARP1 to examine the ADP-ribosylation sites in the PARP1 auto-modification domain (Supplementary Fig. 5). The three ADP-ribosylation sites (D387, E488 and E491) previously identified in the automodification domain^13^ and conserved glutamic residues E407, E448, E456 and E471 of the single BRCT domain are in close vicinity to the Banf1 dimer. However, it should also be noted that serine residues have also been recently identified as ADP-ribosylation sites on PARP1 substrates and upon PARP1 itself, in the presence of the PARP1-interacting protein HPF1 (refs ^9–12^). Three serine residues, S499, S507 and S519 have been confirmed to be sites of auto-ADP-ribosylatyion, within the PARP1 automodification domain close to the unstructured BRCT domain^10^ and the Banf1 dimer is also predicted to bind in close proximity to these residues.

In order to investigate whether Banf1 binds to the NAD^+^-binding domain, as suggested by the modelling, a Flag-PARP1 construct was made with mutations in the residues predicted to interact with Banf1 and that comprise part of the NAD^+^ binding domain, 906–911A. As predicted, mutation of PARP1 906–911A severely impacted the interaction with endogenous Banf1 protein (Fig. 4c). The model also predicted that one side of the Banf1 dimer interacted with the BRCT domain of PARP1 and in support of this, deletion of the BRCT domain completely inhibited the interaction between Banf1 and PARP1 (Fig. 4d). Taking into account the modelling predictions, we next made Flag-Banf1 mutations of D9A and also of two other acidic residues not implicated in binding. As predicted mutation of aspartic acid residue 9 to alanine (D9A) disrupted the Banf1:PARP1 interaction (Fig. 4e, f) but mutation of other acidic residues did not, unless in combination with D9A (Fig. 4e). As expected, ectopic expression of wild type Banf1 caused suppression of PARP1 auto-ADP-ribosylation, while expression of the Banf1 D9A mutant, that displays reduced binding to PARP1, failed to inhibit PARP1 auto-ADP-ribosylation (Fig. 4e, g). This was shown not be due to D9A mislocalisation, as D9A was observed to localise similarly to wild-type Banf1 before and after H_2_O_2_ (Supplementary Fig. 6a, b). These data suggest that Banf1 binds to PARP1 directly and functions to inhibit PARP1 activity.

Since our modelling and mutagenesis data suggested that Banf1 binding may block the entry of NAD^+^ into the binding pocket of PARP1, we investigated this possibility further in vitro. To study the effect of NAD^+^ binding the well-characterised PARP1 E988K mutant was used, which has ~1% of the poly-ADP-ribose activity of wild type PARP1 without affecting NAD^+^ binding^26,27^ and showed no detectable auto-ADP-ribosylation activity in our studies (also observed in Fig. 2d). The presence of Banf1 was able to significantly inhibit the binding of both biotinylated (Fig. 4h and Supplementary Fig. 6c, d) and P32 labelled NAD^+^ to PARP1 (Supplementary Fig. 6e), supporting the prediction that Banf1:PARP1 binding inhibits NAD^+^ binding. In support of this the D9A Banf1 mutant, that displayed reduced binding to PARP1, was unable to block NAD^+^ binding to the same extent as wild-type Banf1 (Supplementary Fig. 6f).

### Mutation of Banf1 A12T inhibits PARP1 activity

A single homozygous point mutation of Banf1 alanine 12 to threonine (A12T) leads to the premature ageing syndrome, Néstor–Guillermo progeria syndrome (NGPS)^6,7^. It was originally asserted that mutation of Banf1 A12T destabilised the Banf1 protein^7^, but we subsequently demonstrated that the A12T mutated Banf1 was stable, and that the mutation reduced the antigenicity of the Banf1 antibody against Banf1, disrupting detection^28^. The original antibody was raised against the N-terminal of Banf1, however, a commercially available Banf1 antibody raised against the C-terminal of the Banf1 protein detects exogenous expression of mutant A12T protein (Supplementary Fig. 7a, b). This C-terminal Banf1 antibody also detected expression of the mutant protein within the NGPS patient cells, including Banf1 localisation to the nuclear envelope (Supplementary Fig. 7c, d). This is significant as the Banf1 A12T mutant protein is expressed to similar levels in patient cells as wild-type Banf1 in normal cells, suggesting that it is the presence of the mutant protein that causes the pathology of the NGPS premature ageing syndrome and not the lack of Banf1 protein.

**Fig. 5:**
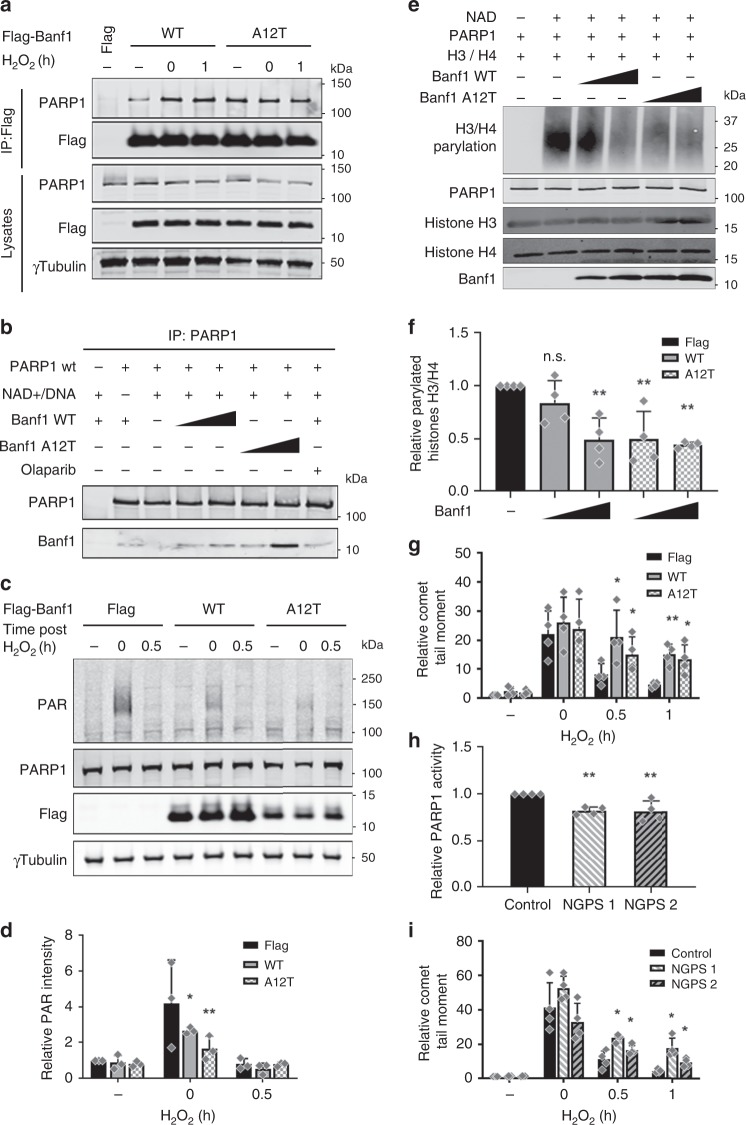
Banf1 A12T inhibits repair of oxidative lesions in NGPS patient cells. **a** Interactions of PARP1 with Flag-Banf1 mutants. Flag immunoprecipitations from HEK293T cells ectopically expressing the Flag-Banf1 WT or A12T proteins following H_2_O_2_. **b** Purified Banf1 WT or A12T proteins were incubated with PARP1, immunopreciptated with PARP1 antibodies and immunoblotted with the indicated antibodies. **c** Inhibition of auto-poly-ADP-ribose activity of PARP1 in U2OS expressing ectopic Flag-Banf1 WT or A12T following H_2_O_2_. **d** The PAR bands in (**c**), were analysed via densitometry and normalised to γ-Tubulin. Histogram data shown in (**d**), represent the mean and S.D. of three independent experiments. **e** In vitro inhibition of PARP1 poly-ADP-ribose activity on histones by purified Banf1 WT or A12T. **f** The PAR bands on (**e**), were analysed via densitometry. **g** Banf1 inhibits repair of oxidative DNA damage. Alkaline comet assay showing the relative olive tail moment in control cells and cells ectopically expressing Flag-Banf1 WT or A12T following H_2_O_2_ treatment and recovery. Paired *t* test was used for statistical analysis. **h** Inhibition of Poly-ADP-ribose activity of PARP1 purified from NGPS patient cells on an immobilised histone substrate HEK293T following H_2_O_2_. **i** NGPS patient cells exhibit defective repair of oxidative DNA damage. Alkaline comet assay showing the relative olive tail moment in control cells and NGPS cells after H_2_O_2_ treatment and recovery. Paired *t* test was used for statistical analysis. Immunoblots are representative of *n* = 3 independent experiments. Unless otherwise stated, histogram data shown represent the mean and S.D. of *n* = 4 independent experiments and statistical significance was defined via ANOVA. *P < 0.05, **P < 0.01. Source data are provided as a Source Data file.

Significantly, in contrast to wild type Banf1, the Banf1 A12T mutant protein was found to interact with PARP1 to the maximal amount in the absence of DNA damage (Fig. 5a, Supplementary Fig. 7e). Banf1 A12T was localised to chromatin, similarly to wild-type Banf1 before and after H_2_O_2_ (Supplementary Fig. 7f, g). It was also observed that purified Banf1 A12T displayed increased binding to PARP1 compared to wild-type Banf1 in in vitro pulldowns (Fig. 5b). The increased interaction in vitro suggests that the increased interaction was probably not due to an increase in DNA damage in A12T expressing cells but was more likely due to a structural change in Banf1 promoting an interaction with PARP1. Consistently, expression of Banf1 A12T significantly reduced the auto-ADP-ribosylation of PARP1 in vivo (Fig. 5c, d) and in vitro histone H3/H4 assays (Fig. 5e, f).

### Repair of oxidative lesions is disrupted in patients with NGPS

To assess the impact of A12T mutation on repair of oxidative lesions we carried out an alkaline comet assay. A12T expression was found to inhibit DNA repair of oxidative lesions to a similar extent as overexpression of wild-type Banf1 (Fig. 5g, Supplementary Fig. 8a). We next obtained two NGPS (Banf1 A12T mutant) patient skin fibroblast cell lines and measured the PARP1 activity in these cell lines. Following oxidative stress PARP1 activity on an immobilised histone substrate was significantly reduced in NGPS cells compared to wild-type cells (Fig. 5h). Both patient cell lines also exhibited defective DNA repair of oxidative lesions as measured by alkaline comet assay (Fig. 5i, Supplementary Fig. 8b). To assess whether the oxidative DNA damage repair defect in the NGPS cell lines was caused by the presence of the Banf1 A12T protein we depleted Banf1 from NGPS patient cells and control cells and carried out comet assay analysis. This confirmed that depletion of Banf1 A12T protein in both NGPS patient cell lines was sufficient to restore repair of oxidative lesions, comparable to control cells (Supplementary Fig. 8c–e). The defective oxidative DNA repair observed in the NGPS patient cells was comparable to a partial depletion of PARP1 protein in control cells. Whereas, expression of a catalytically inactive PARP1 E988K mutant led to a greater repair defect (Supplementary Fig. 8f–h).

Despite binding more robustly, Banf1 A12T was not able to block NAD^+^ binding more efficiently than wild-type Banf1 (Supplementary Fig. 9a), suggesting that the greater inhibition of PARP1 in cells expressing A12T is likely due to another mechanism. In support of this, our modelling studies were unable to detect any difference between WT and A12T: PARP1 binding, with both proteins appearing to bind with similar affinities. In the absence of a higher resolution three-dimensional structure of Banf1:PARP1, we hypothesise that the flexible and disordered BRCT-WGR linker of PARP1 might be allosterically modulating protein-protein interactions with one of the monomers of the Banf1 dimer and that A12T may further promote this interaction, leading to inhibition of PARP1 auto-poly-ADP-ribosylation.

PARP1 protein levels were comparable to control cells in the NGPS patient cells (Supplementary Fig. 9b). PARP1 recruitment following H_2_O_2_ observed in NGPS cells and cells expressing ectopic A12T was comparable to that in wild type cells (Supplementary Fig. 9c, d) and PARP1 was observed to colocalise with ectopically expressed Banf1 A12T (Supplementary Fig. 9d).

The poly-ADP-ribose activity of PARP1 is dependent upon NAD^+^, but the decreased oxidative stress-induced PARP1 activity observed in NGPS cells was not due to lower NAD^+^ levels as NAD^+^ levels were shown to be increased in these cells (Supplementary Fig. 10a). In contrast NAD^+^ levels were significantly decreased in Banf1-depleted cell lines (Supplementary Fig. 10b).

## Discussion

There have been significant advances in our understanding of PARP1 structure and function in recent years, however, the regulatory mechanisms governing PARP1 activity are still largely unknown. The data presented here define a direct role for Banf1 in the regulation of PARP1 ADP-ribose activity.

Here, we have characterised the response of the nuclear envelope protein Banf1 to oxidative stress, which promotes its relocalisation from the nuclear envelope to colocalise with and bind to PARP1. Banf1 binds to the NAD^+^ binding domain of PARP1 and is able to block NAD^+^ from entering the NAD^+^ binding domain of PARP1, preventing PARP1 poly-ADP-ribose activity.

In recent years, several PARP1 regulatory proteins have been identified, including SAM68, HPF1 and YB-1 (refs ^14,29–31^). To the best of our knowledge, all of the known PARP1 regulatory proteins stimulate the activity of PARP1, with the exception of poly-(ADP-ribose) glycohydrolase (PARG)^32^, which removes the poly-ADP-ribose chains from PARP1 and its substrates. In contrast, Banf1 is the first identified PARP1 regulatory protein to directly inhibit PARP1 activity via binding to its NAD^+^ binding domain to block substrate binding.

We envision two possible mechanisms for increased binding of A12T Banf1 to PARP1. We have previously shown using modelling, CD spectra and EMSA studies that A12T mutant does not induce secondary structure change but causes only minor rearrangement of the side-chains disrupting the interaction of Banf1 with DNA, in agreement with the current experimental data^28^. In the first mechanism, as we have shown previously the Banf1 A12T protein is impaired in its ability to bind DNA and since the DNA-binding site and PARP1 binding site within Banf1 share overlapping residues it seems likely that Banf1 A12T would be more available to bind to PARP1 than wild-type Banf1, since a proportion of wild-type Banf1 would also be bound to DNA. However, this model only explains the increased binding in part and does not offer an explanation for the increased binding of Banf1 A12T to PARP1 in vitro, in the absence of DNA.

The second mechanism is based on the molecular model where the NAD^+^ and Banf1 sites within PARP1 are partially overlapping. While the small molecules like NAD^+^ or its benzamide analogues could access both the nicotinamide and the deeper adenosine pocket in an open, unfolded helical subdomain (HD) conformation^33,34^, our study indicates that a macromolecule like Banf1 could only access the nicotinamide binding site. This is supported by our data showing that Banf1 partially blocks NAD^+^ binding to PARP1, but does not completely inhibit it.

We are unable to accurately model the side of the Banf1 dimer, which is involved in binding to PARP1 around the BRCT domain, as this region of PARP1 is unstructured/flexible and the current models available cannot account for the localisation/structure of this region. Since Banf1 A12T did not significantly inhibit NAD^+^ binding to PARP1, we consider it is likely to be the other side of the Banf1 dimer, that would be in proximity with the BRCT domain of PARP1, where A12T would be likely to have a stronger binding and inhibitory effect on PARP1 activity. We anticipate that Banf1 will act to either directly perturb the HD structure, or alter the HD structure by engaging the WGR/BRCT domain. Lastly, due to Banf1 binding in close proximity to the BRCT domain, it could also directly block the auto-ADP-ribosylation of PARP1 residues, required for it’s activity. Understanding the exact nature of this interaction and mechanism will form part of a future study.

PARP1 activity declines with age and its increased expression has been implicated in improved health and longevity^17–19^. Conversely, we also show here that mutation of Banf1 impacts upon PARP1 activity and repair of oxidative lesions in a human progeria syndrome, potentially highlighting the significance of this function for human health. Cells from patients with Werner syndrome and Hutchinson–Guildford progeria also display defects in PARP1 activity and defective DNA repair^35–39^. Therefore, together with the data from NGPS patient cells presented here, it leads us to speculate that this mechanism may contribute, in part, to the overlapping pathologies in these progeroid diseases. However, it is also likely that Banf1 has other uncharacterised roles in the cell that may also promote NGPS patient phenotypes.

We speculate that similarly to our previous work on DBC1 (ref. ^19^), the Banf1-dependent regulation of PARP1 forms another mechanism to enable cellular control of NAD^+^ concentration, preventing the hyperactivation of PARP1 and subsequent depletion of NAD^+^ following DNA damage (Supplementary Fig. 11). This hypothesis is supported by the higher NAD^+^ levels observed in NGPS cells with lower PARP1 activity and lower NAD^+^ levels in Banf1 deficient cells with higher PARP1 activity, although it is important to note that this may not be as a direct result of PARP1 inhibition.

In summary, these data support a model whereby cellular levels of Banf1 are crucial to reset oxidative-stress-induced PARP1 activity. Together, these data offer insight into the Banf1-regulated, PARP1-directed repair of oxidative lesions.

## Methods

### Ethics

All experimental procedures were approved by the Queensland University of Technology; Human Research Ethics Committee (approval numbers 1700000940 and 1900000269).

### Cell lines

The U2OS and HEK293T cells were obtained from CellBank Australia (catalogue numbers 92022711 and 85120602, respectively). U2OS cells were grown in RPMI media, supplemented with 10% foetal bovine serum (FCS), HEK293T cells were grown in DMEM media, supplemented with 10% FCS. The NGPS patient cells and a matched control cell line were a kind gift from Carlos Lopez-Otin and were established from two different patients with NGPS. The NGPS cells and control cell line were grown in DMEM high glucose (Invitrogen 41965-062), supplemented with 10% FCS. Cell lines were grown at 37 °C, 5% CO_2_ and at atmospheric O_2_, unless otherwise stated.

### Chemical reagents

All reagents were purchased from Sigma with the following exceptions; Olaparib was purchased from Selleck Chemicals, Biotinylated NAD^+^ was from R&D Systems and NAD^+^ p32 isotope was purchased from Perkin Elmer. The PARG inhibitor PDD00017273 was used at a concentration of 1 μΜ. The PARP1 inhibitor, BYK204165 was used at a concentration of 10 μΜ.

### Constructs

Myc-DDK-tagged human PARP1 (Flag-PARP1) was purchased from OriGene (RC207085). The BRCT deletion mutant for Flag-PARP1 were generated using a Quickchange II XL Site Directed Mutagenesis kit (Stratagene), and verified by DNA sequencing. The Flag-Banf1 and Flag-PARP1 mutants were synthesised and mutated using site-directed mutagenesis by Genscript in the pcDNA3.1 + N-DYK vector in the BamHI-XhoI cloning sites. These constructs were sequenced using the CMV primer (5′-CGCAAATGGGCGGTAGGCGTG-3′). The His-Banf1 wild-type and mutants were synthesised and mutated by Genscript in the pET-28a(+) vector in the NdeI-XhoI cloning sites. These constructs were sequenced using the T7 primer (5′-TAATACGACTCACTATAGG-3′).

### Antibodies

The antibodies used were as follows: anti-Banf1 N-terminus (SAB1409950, Sigma-Aldrich and ab88464, Abcam, 1:1000 for WB and 1:500 for IF), anti-Banf1 C-terminus (PRS40170604, Sigma-Aldrich, 1:1000 for WB and 1:500 for IF), PARP1 (9532, Cell Signalling Technology 1:1000 for WB and 1:500 for IF), PARP1 (ab191217, Abcam Fig. 2e 1:500 for IF), anti-Emerin (5430, Cell Signalling Technology, 1:500 for IF), anti-Flag M2 Antibody (F3165, Sigma-Aldrich, 1:1000 for WB and 1:300 for IF), anti-PAR (ab14459, Abcam, 1:1000 for WB), anti-γ-Tubulin (T6557, Sigma-Aldrich, 1:2000 for WB), anti-H3 (4499, Cell Signalling Technology, 1:2000 for WB), anti-H4 (2935 Cell Signalling Technology 1:1000 for WB), anti-SP1 (9389, Cell Signalling Technology, 1:1000 for WB), anti-EGFR (sc-03, Santa-cruz, 1:500 for WB), anti-LC3B (2775, Cell Signalling Technologies, 1:1000 for WB), anti-phospho-p53 ser15 (2984, Cell Signalling Technology, 1:1000 for WB), anti-β-actin (612656, BD Biosciences, 1:2000 for WB). Fluorescent secondary antibodies used were: Donkey anti-Mouse 800 nm (LiCor; IRDye 800CW 926-32212, 1:5000 for WB), Donkey anti-Rabbit (LiCor; IRDye 680LT 926-28023, 1:5000 for WB) and Alexa Fluor 488 (Cat# A32766, Molecular Probes, 1:200 for IF) and 594 (Cat# A32754, Molecular Probes, 1:200 for IF).

### siRNA

Control, Banf1 and PARP1 pooled esiRNA were purchased from Sigma. Control and Banf1 siRNA were purchased from GenePharma (Banf1, GGGUUUUGACAAGGCCUAUdTdT). Cells were typically assayed 72 h after transfection.

### ShRNA generation and transformation

ShRNA sequences (Banf1-1 shRNA: GGGAATGGCTGAAA, Banf1-2 shRNA GAATGGCTGAAAGACACTT) were cloned into pLKO.1 (Addgene: 10878) as per Addgene referenced protocol (pLKO.1 TRC cloning).

The day before transfection (Day 1), 293FT cells were plated into a T75 tissue culture plate so that they would be 90–95% confluent on the day of transfection. For each transfection reaction Fugene HD complexes were generated as follows: 36 μl Fugene + 1.5 ml OptiMEM per reaction then added 0.5 μg pTAT, 2.8 μg pHEF-VSV-G and 7.1 μg pNHP + 3.5 μg of Lentiviral Plasmid (pLKO.1 containing shRNA) per reaction. Complexes were incubated for 15 min and added to each plate of cells. Incubate the cells overnight at 37 °C in a humidified 5% CO_2_ incubator. The next day media was replaced. Virus was harvested after 48/72 h by collecting the condition media and centrifuging the 293FT cells.

To transform cells: Polybrene (2 μg/mL) was added to plated U2OS cells, followed by the addition of 1 mL of virus containing media (T25). Cells were cultured for 48–72 h for optimal depletion of Banf1.

### Transfections

All DNA constructs were transfected using Fugene HD (Promega) as per manufacturer’s instructions. RNAiMax (Invitrogen) was used to transfect esiRNA and siRNA as per manufacturer’s instructions.

### Immunoblotting

Cells were lysed (lysis buffer: 20 mM HEPES pH7.5, 250 mM KCl, 5% glycerol, 10 mM MgCl_2_, 0.5% Triton X-100, Protease inhibitor cocktail (Roche) and phosphatase inhibitor cocktail (Cell Signalling)) and sonicated. Lysates were cleared by centrifugation. Typically, 30 μg of protein lysate was separated on a 4–12% sodium dodecyl sulfate (SDS)-polyacrylamide gel electrophoresis (Invitrogen) blocked in Odyssey buffer (LiCor Biosciences) and immunoblotted with the indicated antibodies. Immunoblots were imaged using an Odyssey infra-red imaging system (LiCor).

### Immunoprecipitation

Cells were treated as stated then lysed and sonicated as for immunoblotting. Lysates were transferred to a new tube and incubated with Pierce^™^ Universal Nuclease for Cell Lysis to digest DNA. Digested lysates were then incubated with the indicated antibodies or equivalent amount of IgG for the relevant species for 1 h on rotation at 4 °C. Protein A or G magnetic Dynabeads (Thermofisher) were then added to the tubes and were incubated for 1 h on rotation at 4 °C. Beads were then washed 5 times in lysis buffer and boiled in 2× SDS loading dye.

### Direct interactions

The purified wild type PARP1 and PARP1 E988K recombinant proteins were purchased from Sigma and Abcam, respectively. In order to purify Banf1 recombinant protein, the method was adapted from ref. ^28^. Plasmids expressing HexaHis-tagged WT or A12T Banf1 were transformed into BL21 (DE3) pLys *Escherichia coli*. *E. coli* were grown at 37 °C and 1 mM IPTG was used to induce protein expression for 3 h. Following induction, the *E. coli* were harvested by centrifugation and stored overnight at −80 °C. Cell pellets were lysed in 8 mL of lysis buffer/g of cells (25 mM HEPES pH 7.5, 150 mM NaCl) and sonicated. The resulting cell lysates were centrifuged for 30 min at 17,000 rpm and the supernatant was discarded. The pellet fraction containing HexaHis Banf1 was resuspended in solubilisation buffer (25 mM HEPES pH 7.5, 150 mM NaCl, 25 mM imidazole) containing 6 M guanidinium chloride, and kept for 1 h at 4 °C, under agitation. The lysate was then centrifuged and the supernatant incubated with HIS-Select® Nickel Affinity Gel for 2 h at 4 °C, under agitation. The affinity gel was washed with the solubilisation buffer and the sample was incubated with 10 mM ATP and 5 mM MgCl_2_ for 20 mins at 4 °C. The protein was eluted from the beads in buffer K (20 mM KH_2_PO_4_, pH 7.4, 0.5 mM EDTA, 10% glycerol, 0.01% IGEPAL) complemented with 300 mM KCl and 250 mM Imidazole. 100 mM DTT was added to eluents and incubated for 2 h at 40 °C to reduce any remaining disulphide bonds.

A 10 kDa Microsep^™^ centrifugal device (Pall corporation) was used to concentrate the purified protein to a volume of 250 μL. The purified protein was then loaded onto a Superose 6 10/300 GL size exclusion chromatography column (GE healthcare) and run with K buffer containing 300 mM KCl. Fractions containing monomeric and dimeric Banf1 were pooled, concentrated and stored at −80 °C.

Purified histone H3 and H4 were purchased from NEB. For Banf1:PARP1 interactions, 500 ng PARP1 was incubated with 100 ng wild-type or A12T Banf1 for 5 min in PARP1 activity buffer (50 mM Tris-HCl, pH7.8, 10 mM MgCl_2_ and 1 mM DTT) at room temperature. For experiments where PARP1 was activated prior to interaction, PARP1 was incubated with 250 μM NAD+, 25 μM biotinylated NAD+ (Trevigen) and 25 μg double‐stranded activator oligonucleotide (5′‐GGAATTCC‐3′)^40^ for 2 min at room temperature. PARP1 was then immunoprecipitated and immunoblotted using PARP1 antibodies as above.

### ADP-Ribose measurement via immunoblotting

Cells were treated with 200 μM H_2_O_2_ for 20 min, then cells were scraped and washed once in phosphate-buffered saline (PBS), before immediate lysis in either 4× SDS loading dye (diluted to 1X in lysis buffer following cell lysis) or lysis buffer containing PARG inhibitor (20 mM Hepes pH7.5, 250 mM KCl, 5% glycerol, 10 mM MgCl_2_, 0.5% Triton X-100, 1 μΜ PARG inhibitor (PDD00017273, Sigma), to preserve PAR modifications. Lysates were sonicated before being immunoblotted as before.

### PARP activity assay

PARP1 was immunoprecipitated from the indicated cell lines and activity was determined, according to manufacturer’s instructions, by a Universal Chemiluminescent PARP Assay Kit (Trevigen, # 4676-096-K) based on HRP-streptavidin-mediated detection of biotin-labelled PAR^19^. Luminescence was measured on an EnSpire 2300 Multi-label reader (Perkin Elmer).

### Immunofluorescence

Cells were seeded the day before siRNA transfection. Following siRNA transfection cells were allowed to grow for 48–72 h before treatment or mock-treatment with the indicated DNA damaging agent. Times stated represent time post removal of DNA damaging agents. After treatment cells were treated with an extraction buffer, to remove soluble proteins to enable study of chromatin-bound proteins^41^ for 5 min before fixation in 4% PFA. Cells were permeabilised with 0.2% Triton X-100 for 5 min and blocked in 3% bovine serum albumin for 30 min. Cells were incubated with indicated primary antibodies and Alexa-conjugated secondary antibodies for 1 h each at room temperature. Cells were stained with DAPI, before imaging on a Delta Vision PDV microscope, 60×/1.42 or 100×/1.42 Oil objective (Applied Precision, Inc.). All immunofluorescence figures were assembled using ImageJ. High content imaging was performed using the InCell Analyser 2200 Imaging System (GE Healthcare Life Sciences). Nuclear staining intensity was analysed using the InCell Investigator software (GE Healthcare Life Sciences) with a minimum of 500 nuclei quantified per each independent experiment and the results shown represent the mean and S.D. of three independent experiments. The protein distribution across the cell was measured using ImageJ histogram feature. The average Pearson coefficient, *r*, was calculated from the analysis of 10 cells in each condition using the Image J Coloc 2 analysis software.

### Nuclear envelope quantification

Immunofluorescence was performed as above using anti-Banf1 and anti-Emerin antibodies and images were taken using the personal DeltaVision microscope. Images were analysed using ImageJ, creating a line across the cell and generating histograms (Fig. 1a). To assess the proportion of cells with Banf1 at the nuclear envelope, cells were treated with the indicated treatment and 50 cells in each condition were assessed for the localisation of Banf1 using a Delta Vision PDV microscope.

### Alkaline comet assay

Cells were lifted immediately following mock or 200 μM H_2_O_2_ treatment and 10^3^ cells were mixed with 0.5% low-melting point agarose (Bio-Rad) (37 °C in 1× TBE). The cell suspension was spread onto a comet slide (Trevigen) and immersed in lysis buffer (2.5 M NaCl, 100 mM EDTA, 10 mM Tris (pH10), 1% Triton X-100) for 30 min at 4 °C. Slides were immersed in Alkaline Comet Buffer (300 mM NaOH, 1 mM EDTA) for 30 min before electrophoresis in same buffer at 1 Volt/cm (∼300 mA) for 30 min. Slides were then washed in dH_2_O and dried at 45 °C for 30 min. The DNA was stained using SYBR® Green I (Sigma) (1:10,000) before being dried completely and visualised using a Nikon Eclipse Ti microscope. Quantitation of comet tail moments was performed on a minimum of 50 cells using ImageJ plugin where the densitometry of the head and tail, as well as length were measured to calculate the comet tail moment. Assays were performed using at least three biological repeats. Results are displayed as mean ± S.D.

### Subcellular fractionation

To detect binding of proteins to chromatin, subcellular fractionation was carried out using a Subcellular Protein Fractionation Kit for Cultured Cells, according to the manufacturer’s instructions (Thermo Fisher Scientific).

### Protein modelling

Protein–protein docking: Several crystal structures of PARP1 inhibitors based on derivatives of natural compounds, such as nicotinamide adenine dinucleotide (NAD^+^) are available, however, there is no crystal structure of the complex of the complex of human PARP1 with NAD+. Molecular modelling study and crystal structure of chicken PARP1 with an NAD+ analogue, CARBA-NAD overlayed with the human sequence (Fig. 3a). The human and chicken PARP1 NAD^+^ binding domain structures are 100% identical. This showed the indicated role of residues Tyr986, His826, Gly863, Lys903, Ser904, Tyr907 and the residues 883–893 consisting of a loop^42,43^. These residues were defined as active site to study interactions between Banf1 and PARP1. The catalytic domain of PARP1 (pdb code 4DQY; chain C)^44^ and Banf1 dimer (Pdb code 2BZF)^45^ were submitted to a protein–protein docking evaluation using the ClusPro2.0 server^46^. This server performs rigid-body docking to sample billions of conformations, a RMSD clustering method to find highly populated low-energy clusters and an energy minimisation refinement to remove steric clashes. The figures were generated with UCSF Chimera^47^. The molecular interface analysis was performed using PDBSUM and PDBePISA.

Decoy analysis: The representative structures of the obtained clusters from Cluspro docking were ranked with CONSRANK^48^. The interface in the docking decoys were analysed, visualised and compared by the COCOMAPS^49^ web tools.

Modelling PARP1 assembly: To provide insight into poly(ADP-ribose) binding and sites of potential modification by PARP-1 and their effect on PARP1–Banf1 complex, we also generated assembly of full-length PARP1. The full-length PARP1 structure was generated using structures of known domains and CORAL programme as previously reported in a SAXS study^50^.

MD simulations of PARP1–Banf1 complex: To test the overall stability of the docked complex, we performed a 100-ns long MD simulation. MD simulation was performed using pmemd.cuda in AMBER16 (ref.^51^) with ff14SB^52^ force field for the protein complex in presence of TIP3P^53^ explicit water molecules and ions with a triclinic box. The closest distance between any atom originally present in solute and the edge of the periodic box was set to 10 Å. The particle mesh Ewald^54^ was used to treat the long-range electrostatic interactions. The nonbonded interactions were truncated with 10 Å cutoff. Periodic boundary condition was imposed on the system during the calculation of nonbonded interactions. The time-step was set at 2 fs and SHAKE was used to constrain the bonds involving hydrogen atom. Langevin thermostat with the collision frequency 2.0 was applied to control the temperature at 300 K. First, the system was minimised with protein constrained to equilibrate the solvent. Second, the system was minimised with protein backbone constrained to equilibrate the amino acid side chains and the solvent. To avoid unnecessary structural drift, we restrained protein Cα atom with 10 kcal/mol Å^2^. Third, protein was released to minimise the whole simulation system. Fourthly, the system was slowly heated to 300 K, followed by a 10 ns equilibration of the whole system in an NPT ensemble at an interval of every 10 fs. Finally, MDCOns^55^ analysis was carried out on the last 90-ns production run to study the conservation of inter-residue contacts during a MD simulation.

### NAD^+^ quantification

NAD^+^ quantification was conducted using an NAD/NADH quantification kit (BioVision, Milpitas, CA) as per the manufacturer’s instructions and normalised to soluble protein content.

### Biotinylated NAD^+^-binding assays

U2OS cells were transfected with Flag-PARP1 E988K. Twenty-four hour after transfection cells were lysed in 150 mM NaCl, 1 mM EDTA, 1 mM EGTA, 1% Triton X-100, 0.5% NP-40, 10 mM Tris HCl, pH 7.4 supplemented with protease inhibitors cocktail cOmplete tablets (Roche) and Cell Signalling phosphatase cocktail, followed by sonication. Fifty microlitre of lysate was added to a Flag M2 96 well plate (Genscript) and incubated at room temperature for 30 min. Wells were then washed three times in lysis buffer. Totally, 1.2 µg of purified Banf1 protein was then added to relevant wells for 30 min at room temperature. Two microlitre of 250 nM biotinylated NAD^+^ (Trevigen) and activating DNA oligo was added to the wells for 10 min at room temperature, followed by 2 washes in lysis buffer, one wash in PBS-0.1% Triton X-100 and one wash in PBS. Streptavidin-HRP antibody was added (Trevigen 1:500) for 30 min, followed by one wash in PBS-0.1% Triton X-100 and two washes in PBS. Peroxyglo reagent was added to each well and the chemo-illuminescence was read on a plate-reader.

### ^32^P-NAD^+^-binding assays

Purified PARP1 E988K was incubated with purified Banf1 protein for 30 min in PARP1 activity buffer (50 mM Tris (pH 8), 150 mM KCl, 12 mM NaCl, 2 mM MgCl_2_, 5 mM DTT, 0.1% Triton X-100. 250 nM ^32^P-NAD^+^ (250 µCi) and activating DNA oligo were added for 5 min. Reactions were passed through G25 spin columns to remove unbound NAD^+^ and total counts were read on a scintillation counter.

### Statistical analysis

Histograms represent the average value ±standard error of the mean. Statistical analysis of the results was made using Prism software (GraphPad). ANOVAs (one-way and two-way) and *t* test (two-tailed) were used for statistical analysis. Data are presented as means and  S.D. from  at least three independent experiments (unless otherwise stated). Statistical significance is represented by *P value < 0.05; **P value < 0.01; ***P value < 0.001; ****P value < 0.0001.

### Reporting summary

Further information on research design is available in the Nature Research Reporting Summary linked to this article.

## Supplementary information

Supplementary Information

Reporting Summary

## Data Availability

The source data underlying Figs. 1b, d, 2a–d, 3, 4c–h, 5 and Supplementary Figs. 1c–e, 2, 3, 4a, c, d, 6b–f, 7a, c, e, f, 8c, d, f–h, 9a, b, 10 are provided as a Source Data file. All data are available from the authors upon reasonable request.

## Source Data

Source Data
