# Supplementary material for: Barrier-to-autointegration factor 1 (Banf1) regulates poly [ADP-ribose] polymerase 1 (PARP1) activity following oxidative DNA damage
Source: Nat Commun. 2019 Dec 3;10:5501. doi: 10.1038/s41467-019-13167-5 (PMC6890647; doi:10.1038/s41467-019-13167-5)
Supplement: Supplementary file 1 — Supplementary Information [file 41467_2019_13167_MOESM1_ESM.pdf]

Barrier-to-Autointegration Factor 1 (Banf1) regulates Poly [ADP-ribose] Polymerase 1 (PARP1) activity following oxidative DNA damage.

Bolderson et al.

Supplementary Information

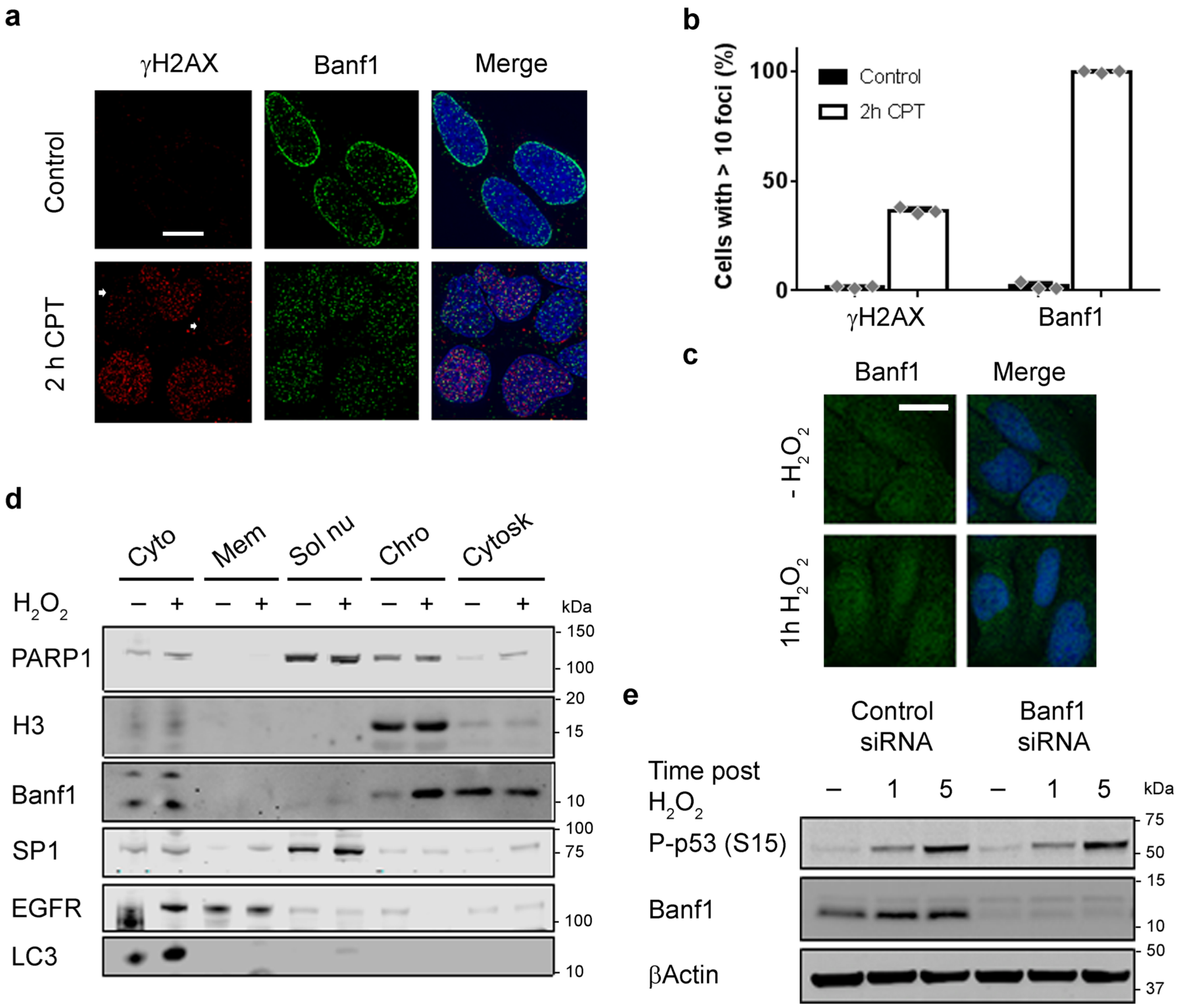

Supplementary Figure 1 | Banf1 is recruited to chromatin following single-strand breaks and oxidative stress. a, Cells were pre-extracted to remove soluble proteins, fixed 2 h post-camptothecin (CPT) and stained with the indicated antibodies. **b**, Cells positive for over 10 Banf1 or γ-H2AX foci were scored as positive 2 h post- camptothecin. **c**, U2OS cells were treated with H_2O_2 and cells fixed (without pre-extraction) 1 hour post- H_2O_2 removal and stained with Banf1 antibodies. **d**, U2OS cells were treated with H_2O_2 and cells lysed 1 hour post-H_2O_2 removal. Lysates were fractionated using a fractionation kit and immunoblotted with the indicated antibodies. **e**, U2OS cells were treated with 200 μM H_2O_2 for 20 minutes and lysates taken at the indicated time points post H_2O_2 removal. Lysates were immunoblotted with the indicated antibodies. The scale bars represent 10 μm. Histogram data shown represent the mean and S.D. of 3 independent experiments (n=3). Source data are provided as a Source Data file.

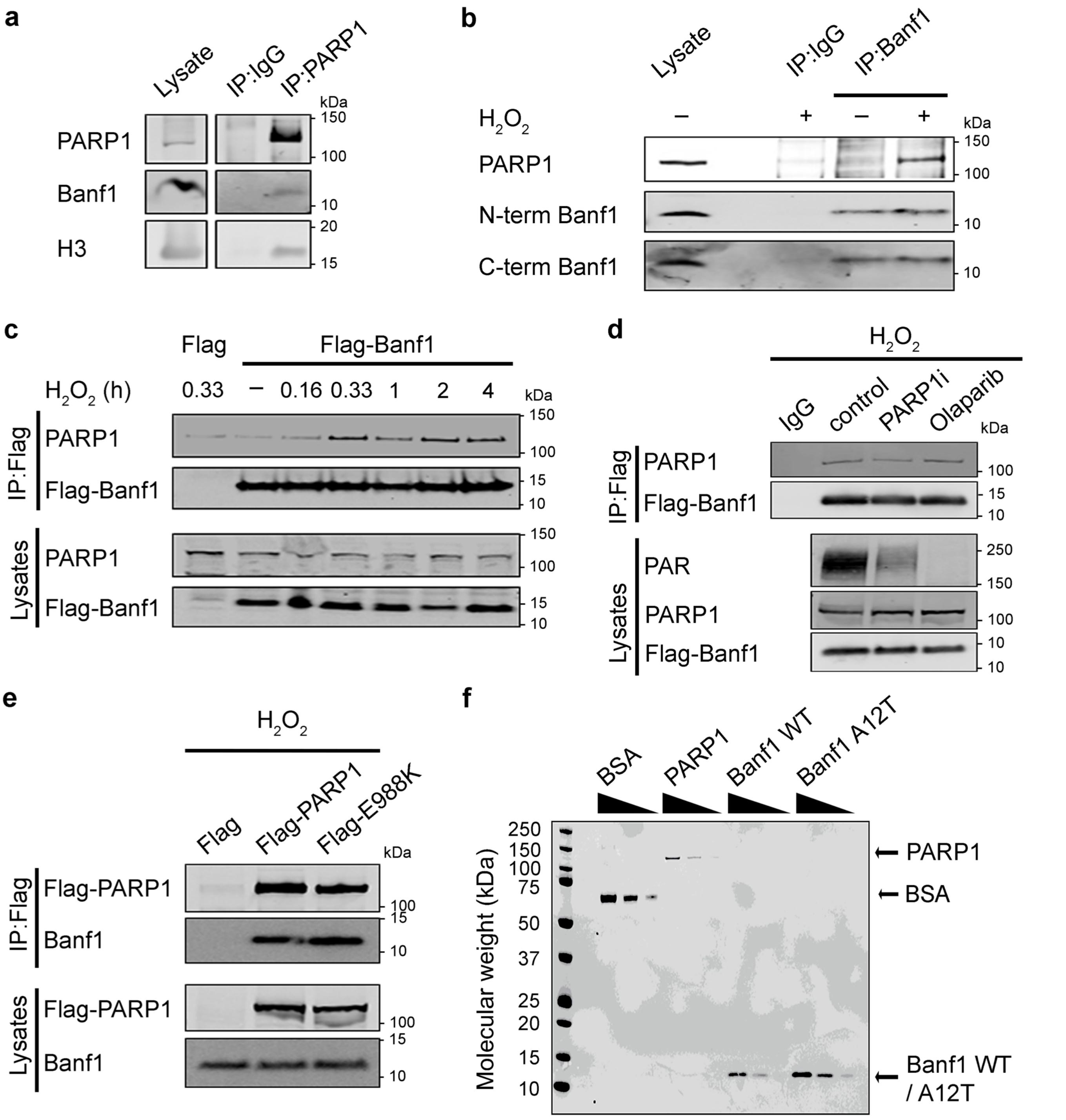

Supplementary Figure 2 | The Banf1:PARP1 interaction is DNA damage-inducible and independent of PARP1 activity. **a**, Lysates from U2OS cells were immunoprecipitated with PARP1 antibodies. Immunoprecipitates were immunoblotted with the indicated antibodies. **b**, Human fibroblasts (AG10803) were mock treated or treated with 200 μM H_2O_2, 1 hour post-H_2O_2 removal, cells were lysed and immunoprecipitated with Banf1 antibodies. Immunoprecipitates were immunoblotted with the indicated antibodies. **c**, HEK293T cells were transfected with Flag or Flag-Banf1 for 24 hours before being mock treated or treated with 200 μM H_2O_2. The first two time points (0.16h and 0.33h) are in the presence of H_2O_2, whereby after 20 minutes treatment H_2O_2 was removed and the time indicated represents the time after H_2O_2 addition. After the indicated times cells were lysed and immunoprecipitated with Flag antibodies. Immunoprecipitates were immunoblotted with the indicated antibodies. **d**, HEK293T cells were treated with 1 μM olaparib or 10 μM specific PARP1 inhibitor, (BYK204165) for 1 hour and mock treated or treated with 200 μM H_2O_2, immediately after treatment, an aliquot of each sample was removed and lysed to retain PAR activity, to measure inhibitor efficiency. The remaining cells were washed and PARP inhibitors replaced. 1 hour after treatment removal the remaining cells were lysed and precipitated with Flag antibodies. Immunoprecipitates were immunoblotted with the indicated antibodies. **e**, HEK293T cells were transfected with Flag, Flag-PARP1 or Flag-PARP1 E988K for 24 hours before being mock-treated or treated with 200 μM H_2O_2, after the indicated times following H_2O_2 removal cells were lysed and immunoprecipitated with Flag antibodies. Immunoprecipitates were immunoblotted with the indicated antibodies. **f**, Purified proteins used in pull-down assays. Purified proteins were run on an 4-12% SDS PAGE gel. The gel was stained with Coomassie Blue and destained before imaging. Western blots are representative of n=3 independent experiments. Source data are provided as a Source Data file.

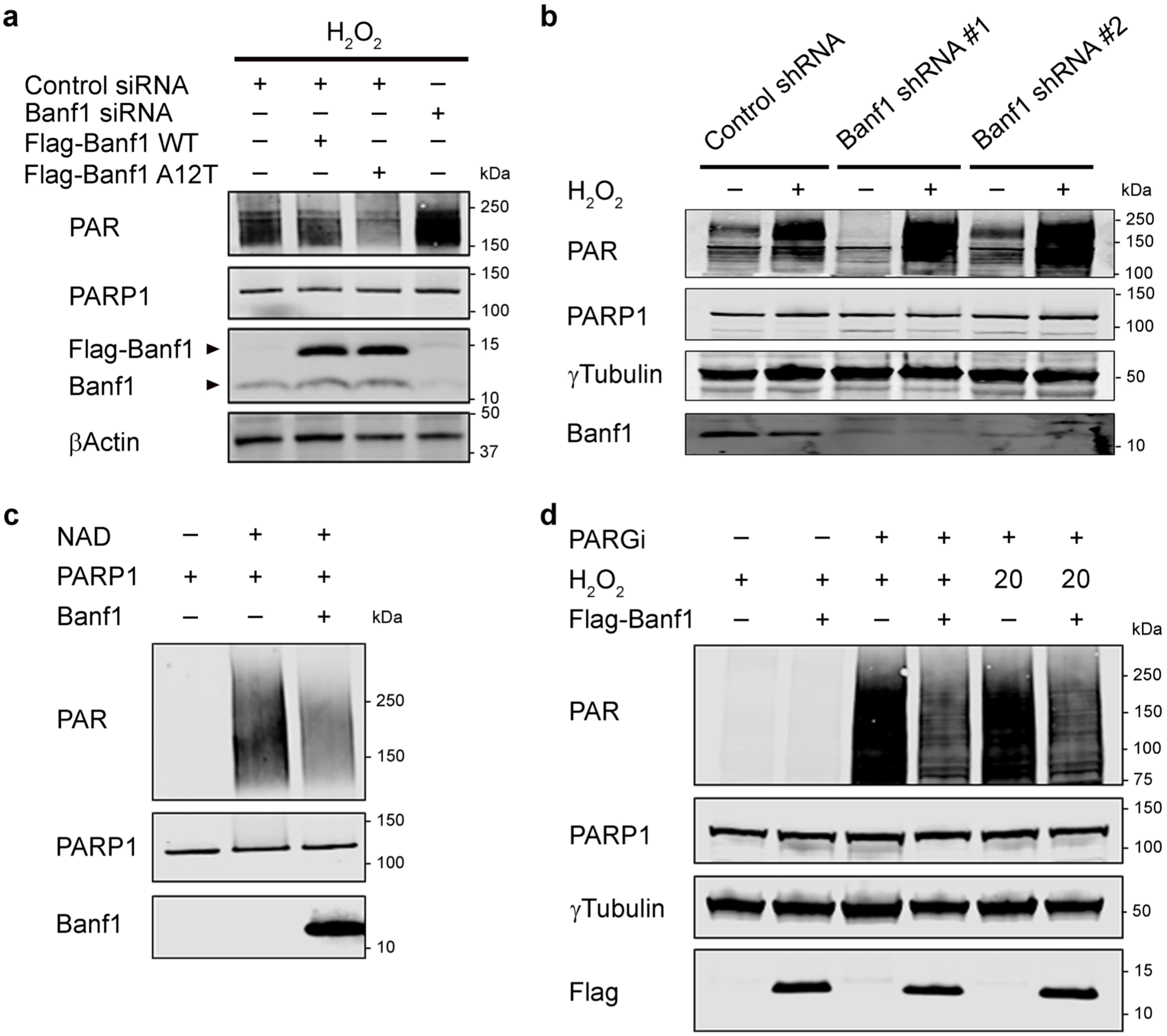

Supplementary Figure 3 | Banf1 depletion increases PARP1 activity following H_2O_2. a, U2OS cells were transfected with control or Banf1 siRNA (Genepharma) for 72 hours before exposure to 200 μM H_2O_2 for 20 minutes. Cells were immediately lysed and lysates were immunoblotted with the indicated antibodies. **b**, U2OS cells were infected with virus expressing Banf1 shRNA and incubated for 72 hours before exposure to 200 μM H_2O_2 for 20 minutes. Cells were immediately lysed and lysates were immunoblotted with the indicated antibodies. **c**, Purified proteins were incubated for 5 minutes (-/+ NAD/DNA) and immunoblotted with the indicated antibodies. **d**, HEK293T cells were transfected with Flag or Flag-Banf1 expression constructs for 24 hours, before mock-treatment or addition of 1 μM PARG inhibitor for 1 hour before incubation with 200 μM H_2O_2 for 20 minutes. Cells were lysed immediately following H_2O_2 treatment and 20 minutes post- H_2O_2 removal. Lysates were immunoblotted with the indicated antibodies. Western blots are representative of 3 independent experiments (n=3). Source data are provided as a Source Data file.

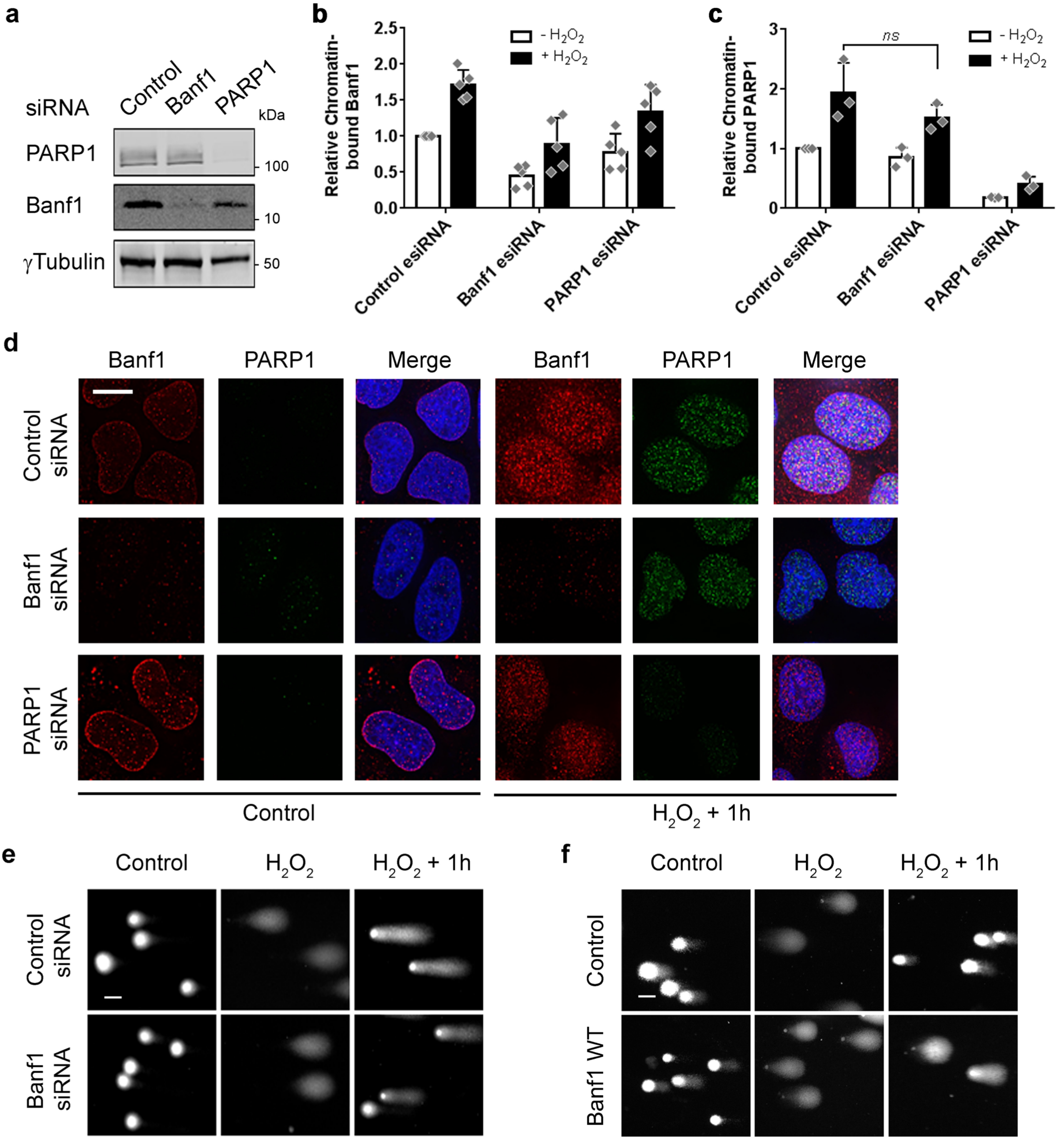

Supplementary Figure 4 | Banf1 and PARP1 localization following H_2O_2. a, U2OS cells were transfected with the indicated esiRNAs for 72 hours. Cells were lysed and lysates were immunoblotted with the indicated antibodies. **b-d**, Cells depleted of Banf1 or PARP1 as in **a**, were seeded into a 96 well plate, exposed to 200 μM H_2O_2 for 20 minutes. 1 hour post-treatment soluble proteins were extracted and cells were fixed. Cells were stained with the indicated antibodies, **d**. The scale bar represents 10 μm. The nuclear intensity of chromatin-bound Banf1, **c**, or PARP1, d, was analysed using an Incell Analyzer 2200. **e-f**, Representative comet assay images showing the relative olive tail moment in control or Banf1 siRNA transfected cells, **e** or cells overexpressing Banf1, **f**. Histogram data represent the mean and S.D. of n=5 and n=3 independent experiments in **c** and **d**, respectively. Source data are provided as a Source Data file.

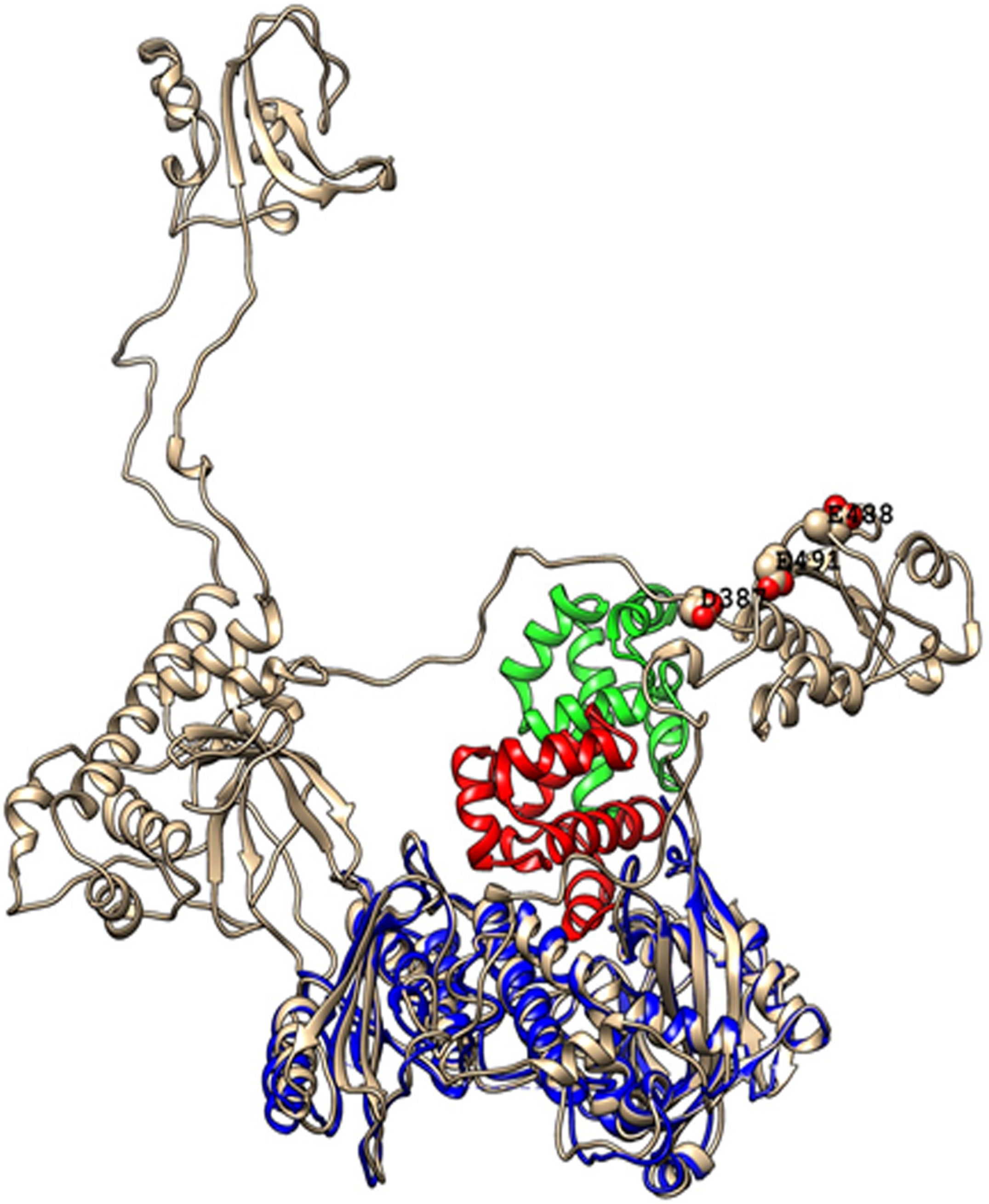

Supplementary Figure 5 | Banf1 binds to the NAD⁺ binding domain of PARP1. Superimposition of a model of full-length Chicken PARP1 (shown in gold) with the docked complex of catalytic domain of Human PARP1 (shown in blue) and Banf1 (shown in green and red). The three ADP-ribosylation sites (D387, E488, and E491) in automodification domain of PARP1 are shown as spheres. These sites are in close vicinity to Banf1.

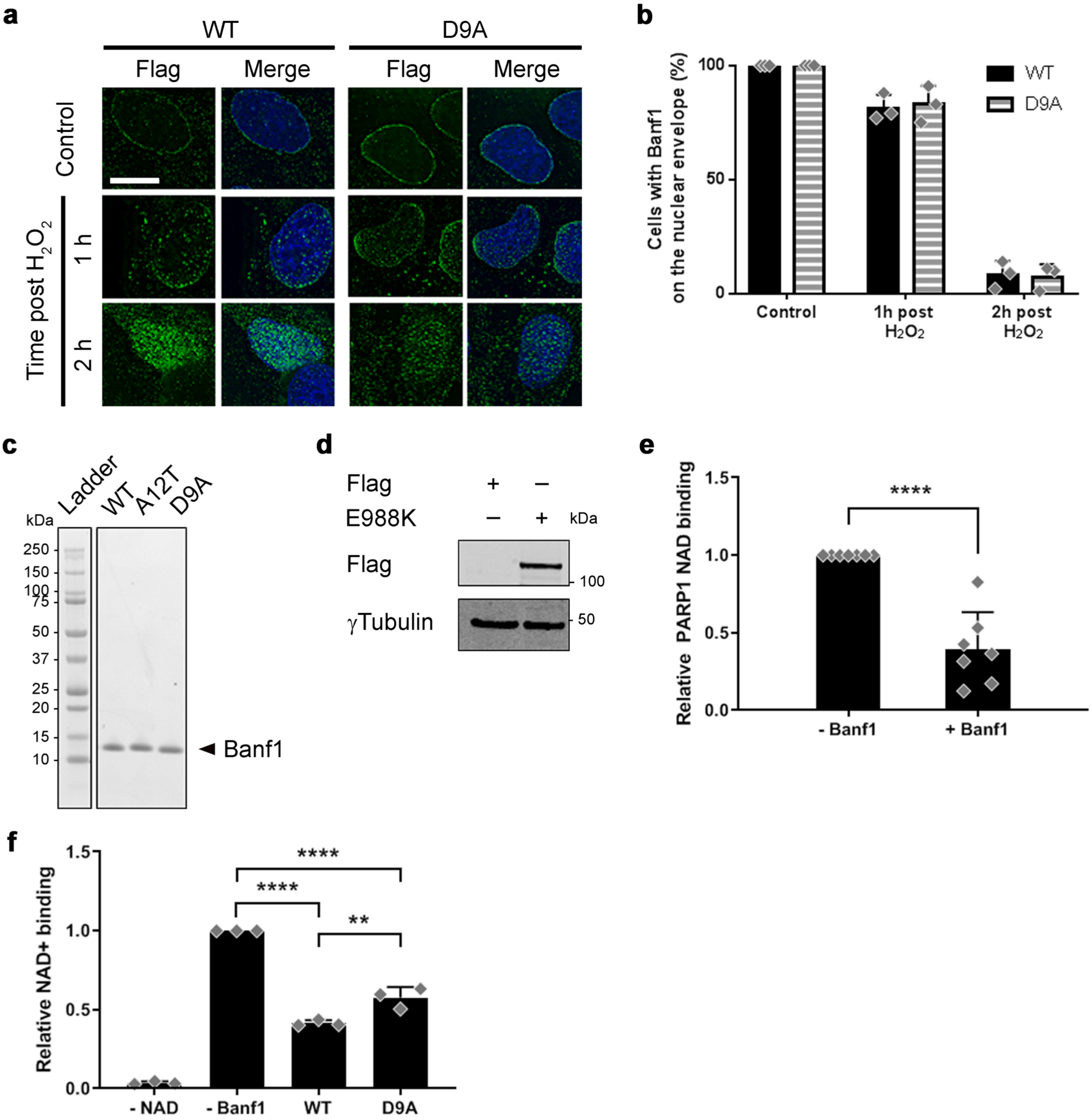

Supplementary Figure 6 | Banf1 binds to the NAD⁺ binding domain of PARP1. a, Banf1 D9A localises similarly to WT Banf1 prior to and following oxidative stress induced by H_2O_2. Cells were pre-extracted and fixed at the indicated times following treatment and stained with Flag antibodies. **b,** Analysis of Banf1 WT and D9A localisation following the indicated times post H_2O_2 removal. 50 cells were analysed in each condition, in 3 independent experiments (n=3). **c**, Purified Banf1 proteins used in NAD binding assays. Purified proteins were run on an 4-12% SDS PAGE gel. The gel was stained with Coomassie Blue and destained before imaging. **d**, Expression of Flag PARP E988K, used in the NAD+ binding experiments. **e**, Purified Banf1 and PARP1 proteins were incubated together for 10 minutes before addition of DNA and P32-labelled NAD⁺. Total counts were measured on a scintillation counter. Histogram data shown represent the mean and S.D. of n=7 independent experiments. **f**. *In vitro* inhibition of PARP1 binding NAD⁺ in the presence of purified WT and D9A Banf1. Unless otherwise stated, histogram data shown represent the mean and S.D. of n=3 independent experiments. Source data are provided as a Source Data file. **P < 0.01, ****P<0.0001.

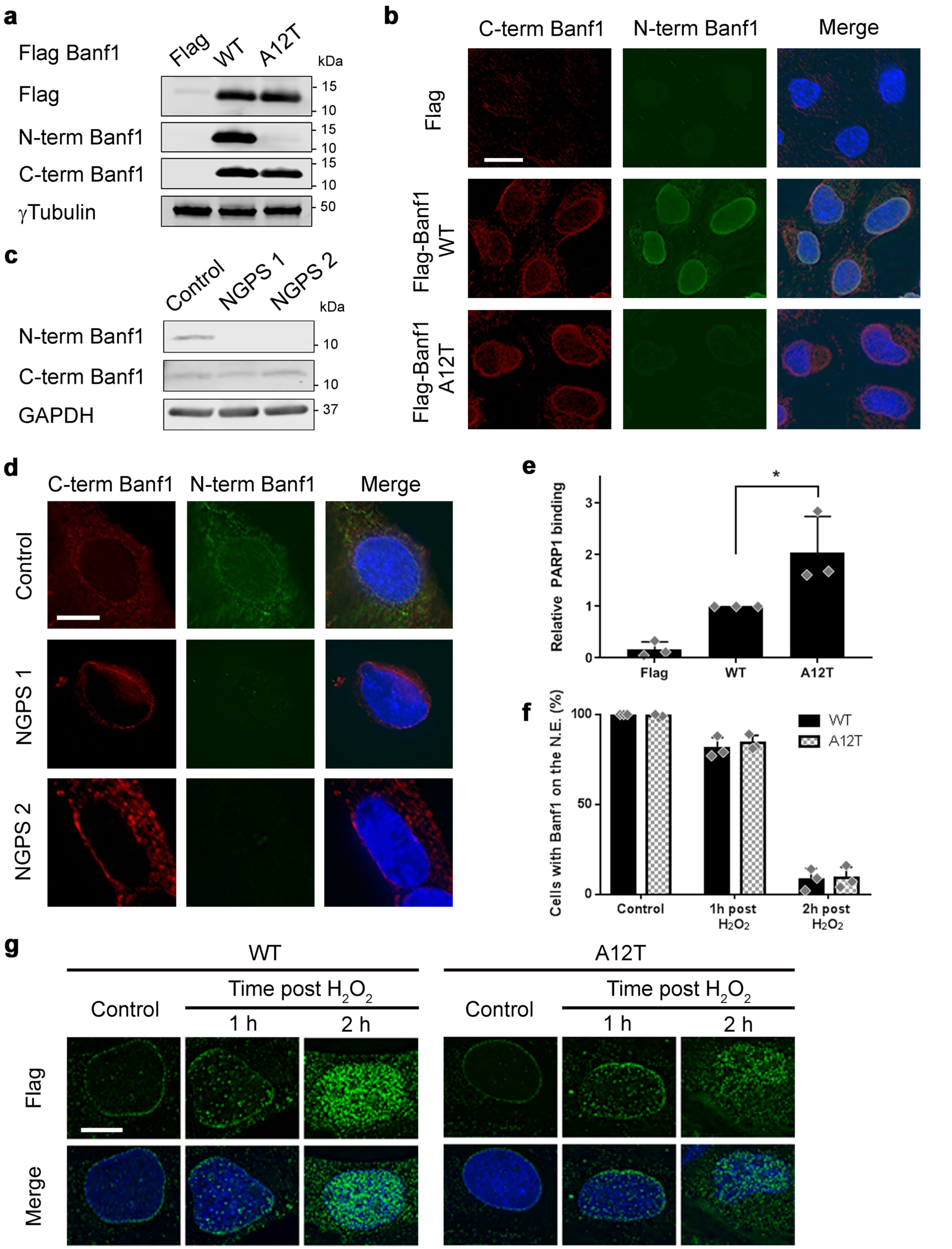

Supplementary Figure 7 | Banf1 A12T mutant localises to the nuclear envelope and can be detected in NGPS patient cells. U2OS cells were transfected with Flag or Flag-Banf1 expression constructs for 24 hours before lysates were taken for immunoblotting, **a**, or immunofluorescence, **b**, with the indicated antibodies. The scale bar represents 20 μm. NGPS cell lysates were taken for immunoblotting, **c**, or immunofluorescence, **d**, with the indicated antibodies. The scale bar represents 10 μm. **e**, Analysis of the relative Banf1 WT vs A12T binding to PARP1 via immunoprecipitation. **f**. Analysis of Banf1 WT and A12T localisation following the indicated times post H_2O_2 removal. 50 cells were analysed in each condition, in 3 independent experiments (n=3). **g**, Banf1 A12T localises similarly to WT Banf1 prior to and following oxidative stress induced by H_2O_2. Cells were pre-extracted and fixed at the indicated times following treatment and stained with Flag antibodies. Histogram data shown represent the mean and S.D. of n=3 independent experiments *$P < 0.05$. Source data are provided as a Source Data file.

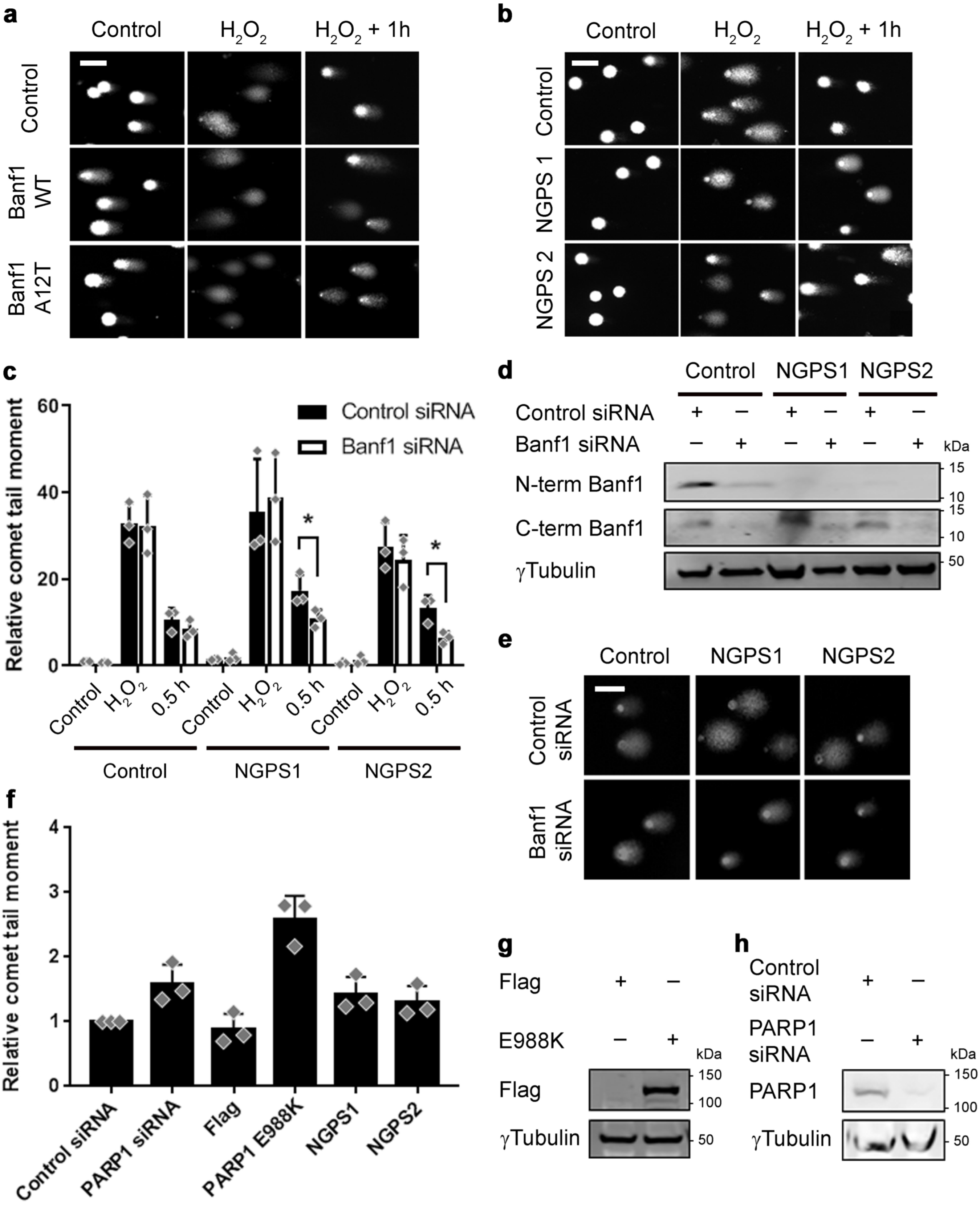

Supplementary Figure 8 | DNA repair of oxidative lesions is repressed in NGPS cells and cells overexpression Banf1 WT and A12T. a, b, Representative comet assay images showing the relative olive tail moment in cells overexpressing WT Banf1 or A12T, **a**, or NGPS cells **b**. The scale bar represents 20 μm. **c,** The defective repair of oxidative damage in NGPS patients can be corrected by depletion of Banf1 A12T, using Banf1 siRNA. Alkaline comet assay showing the relative olive tail moment in control cells and NGPS cells transfected with control or Banf1 siRNA after H_2O_2 treatment and recovery. **d**, immunoblot of cells used in c, showing Banf1 expression and knockdown. **e**, Representative images from c. **f**, The defective repair of oxidative damage in NGPS patient is comparable to partial PARP1 depletion with siRNA. Alkaline comet assay showing the relative olive tail moment in control cells transfected with control or PARP1 siRNA or catalytically inactive PARP1 (E988K) compared with NGPS patient cells, 0.5 h post H_2O_2 treatment and recovery. **g**, Expression of Flag-PARP E988K in the control cells in f. **h**, Depletion of PARP1 using siRNA in the control cells in f. Unless otherwise stated, histogram data shown represent the mean and S.D. of n=3 independent experiments. Source data are provided as a Source Data file. *P < 0.05.

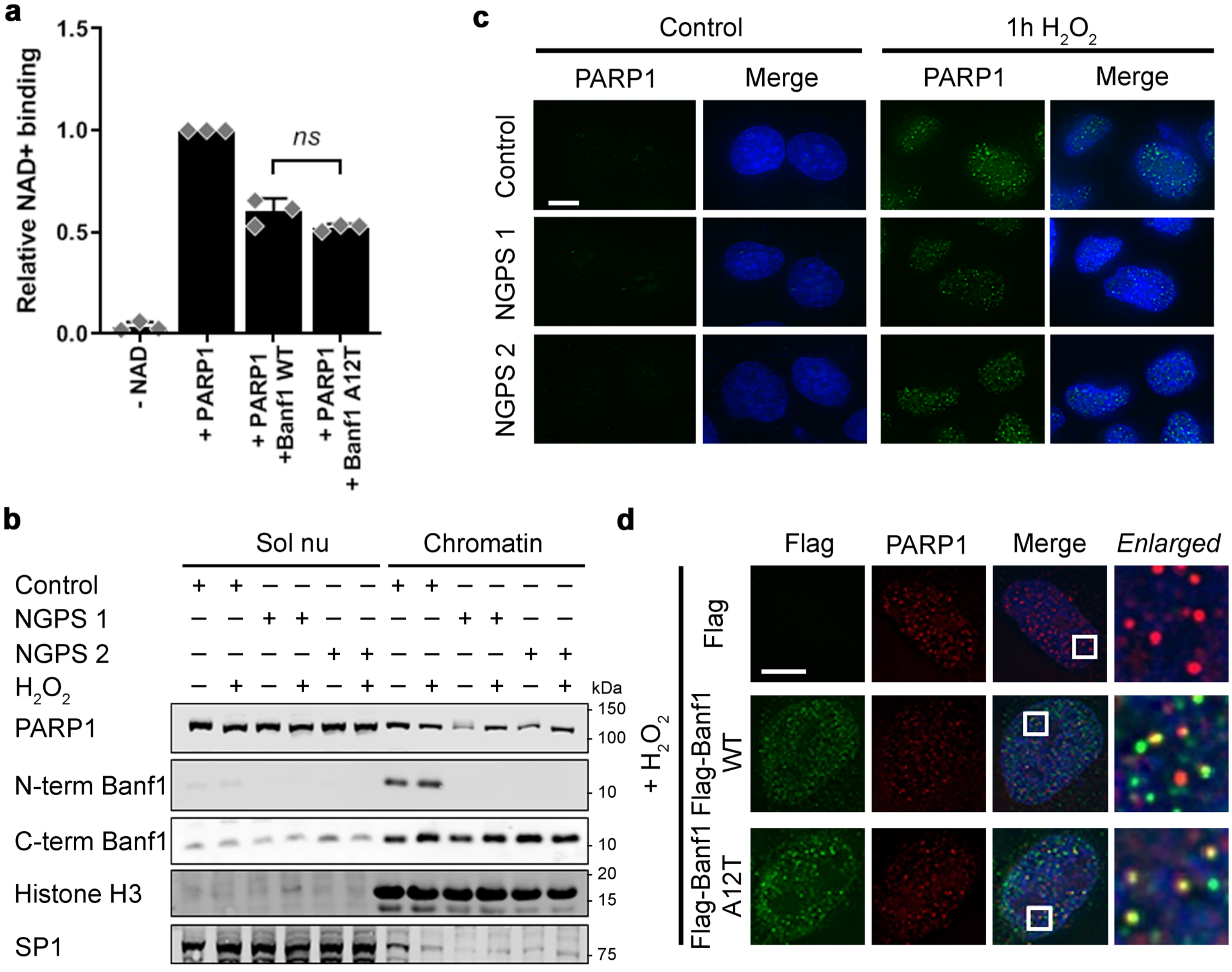

Supplementary Figure 9 | PARP1 is localised normally in NGPS cells or cells expressing Banf1 A12T mutant. a, *In vitro* inhibition of PARP1 binding NAD^+ in the presence of purified WT and A12T Banf1. **b**, NGPS and control cells were treated with 200 μM H_2O_2 and lysates taken 1 hour post- H_2O_2. Lysates were fractionated using a fractionation kit and immunoblotted with the indicated antibodies. **c**, NGPS and control cells were treated with 200 μM H_2O_2 and fixed and stained with the indicated antibodies 1 hour post- H_2O_2. **d**, U2OS cells were transfected with Flag or Flag-Banf1 expression for 24 hours before immunofluorescence with the indicated antibodies. The scale bar represents 10 μm. Unless otherwise stated, histogram data shown represent the mean and S.D. of n=3 independent experiments. Source data are provided as a Source Data file.

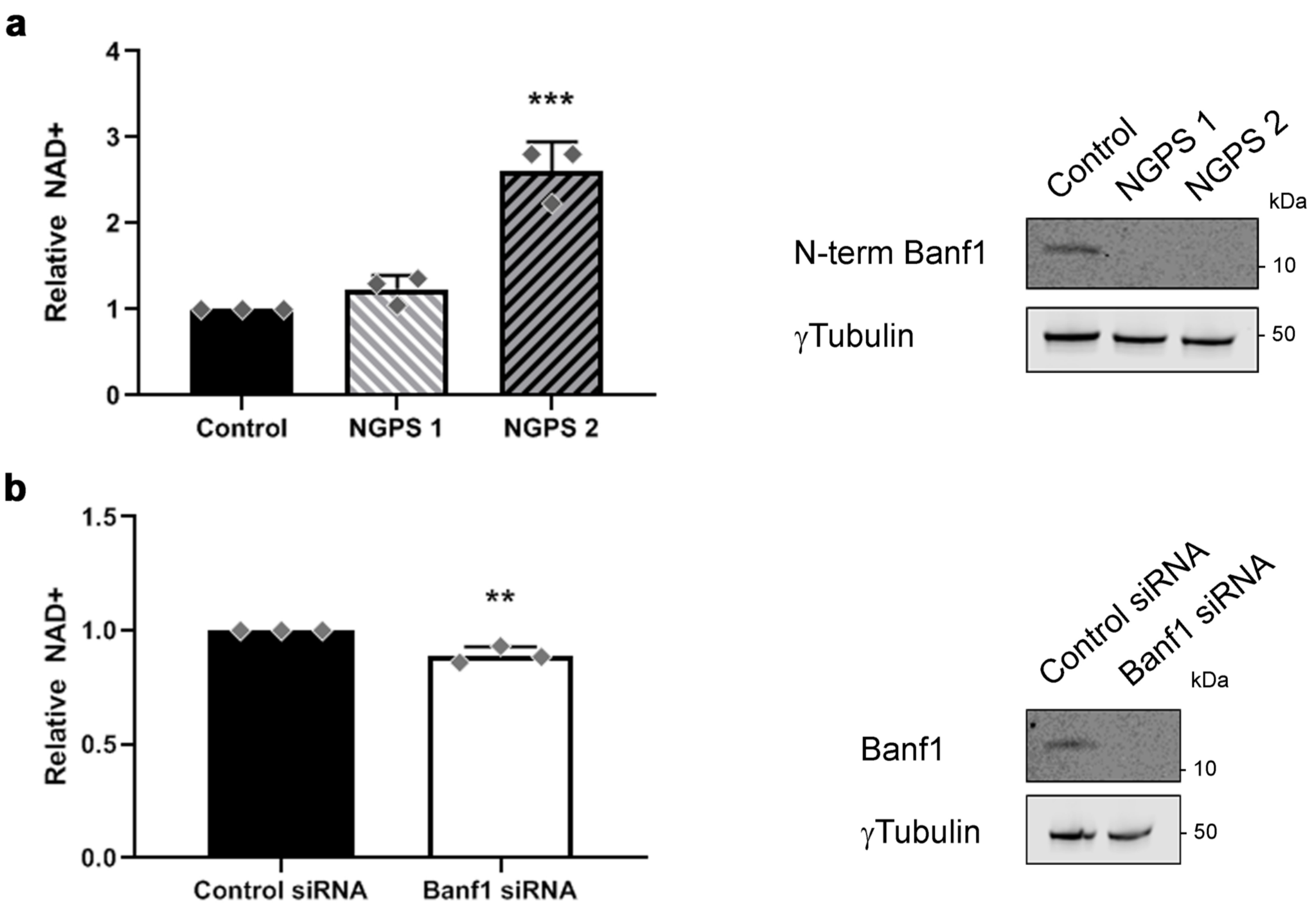

Supplementary Figure 10 | NAD⁺ levels are altered in NGPS cells and Banf1-depleted cells. NAD+ levels in, **a**, NGPS cells and **b**, Banf1 depleted cells. Histogram data shown represent the mean and S.D. of n=3 independent experiments. Representative immunoblots are shown for protein expression. Source data are provided as a Source Data file. **P < 0.01, ***P < 0.001.

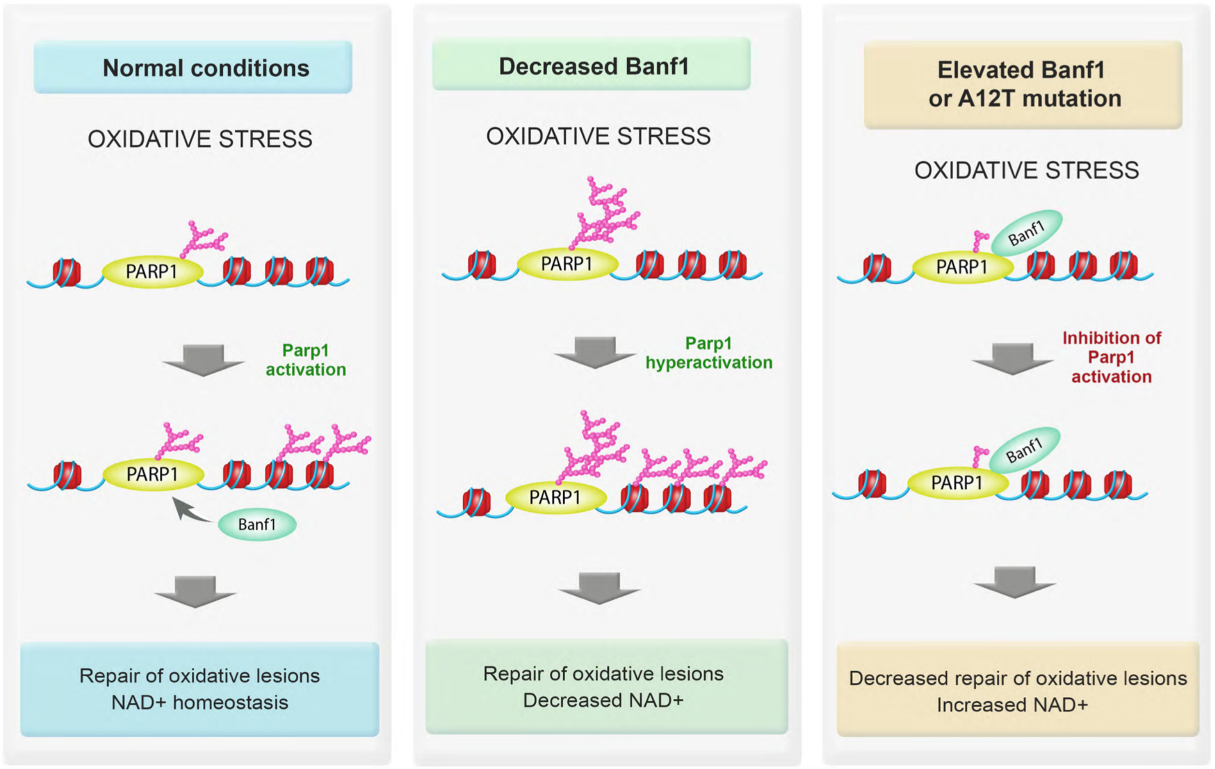

Supplementary Figure 11 | Model for Banf1-dependent regulation of PARP activity. Under normal conditions, PARP1 is activated following oxidative stress and undergoes auto-ADP-ribosylation and ADP-ribosylates its substrates, such as histones. Following repair/recruitment of repair proteins, Banf1 then binds to PARP1 restricting its activity, preventing PARP1 hyperactivation and maintaining NAD^+ homeostasis. When Banf1 is depleted, PARP1 activity is not restrained, hyperactivation occurs following oxidative stress and leads to disruption of NAD^+ homeostasis. In the presence of elevated Banf1 levels or A12T mutation, PARP1 activity is inhibited by binding of Banf1, leading to defective repair of oxidative lesions and increased NAD^+ levels. We propose that this mechanism enables cellular control of NAD^+ concentration, preventing the hyperactivation of PARP1 and subsequent depletion of NAD^+ following DNA damage.
