# Supplementary material for: Barrier-to-autointegration factor 1 (Banf1) regulates poly [ADP-ribose] polymerase 1 (PARP1) activity following oxidative DNA damage
Source: Nat Commun. 2019 Dec 3;10:5501. doi: 10.1038/s41467-019-13167-5 (PMC6890647; doi:10.1038/s41467-019-13167-5)
Supplement: Supplementary file 2 — Reporting Summary [file 41467_2019_13167_MOESM2_ESM.pdf]

nature research

Corresponding author(s): Emma Bolderson and Derek Richard

Last updated by author(s): Oct 11, 2019

Reporting Summary

Nature Research wishes to improve the reproducibility of the work that we publish. This form provides structure for consistency and transparency in reporting. For further information on Nature Research policies, see Authors & Referees and the Editorial Policy Checklist.

Statistics

For all statistical analyses, confirm that the following items are present in the figure legend, table legend, main text, or Methods section.

n/a	Confirmed	
☐	☒	The exact sample size (*n*) for each experimental group/condition, given as a discrete number and unit of measurement
☐	☒	A statement on whether measurements were taken from distinct samples or whether the same sample was measured repeatedly
☐	☒	The statistical test(s) used AND whether they are one- or two-sided<br>*Only common tests should be described solely by name; describe more complex techniques in the Methods section.*
☐	☒	A description of all covariates tested
☐	☒	A description of any assumptions or corrections, such as tests of normality and adjustment for multiple comparisons
☐	☒	A full description of the statistical parameters including central tendency (e.g. means) or other basic estimates (e.g. regression coefficient) AND variation (e.g. standard deviation) or associated estimates of uncertainty (e.g. confidence intervals)
☐	☒	For null hypothesis testing, the test statistic (e.g. *F*, *t*, *r*) with confidence intervals, effect sizes, degrees of freedom and *P* value noted<br>*Give P values as exact values whenever suitable.*
☒	☐	For Bayesian analysis, information on the choice of priors and Markov chain Monte Carlo settings
☒	☐	For hierarchical and complex designs, identification of the appropriate level for tests and full reporting of outcomes
☐	☒	Estimates of effect sizes (e.g. Cohen's *d*, Pearson's *r*), indicating how they were calculated

Our web collection on statistics for biologists contains articles on many of the points above.

Software and code

Policy information about availability of computer code

Data collection	For immunofluorescence analysis, Softworx Version 7 was used on a Delta Vision Microscope.
Data analysis	The ClusPro2.0 server was used to analyse protein:protein interactions.The protein:protein interaction figures were generated with UCSF Chimera39. The molecular interface analysis was performed using PDBSUM and PDBePISA. Decoy analysis: The representative structures of the obtained clusters from Cluspro docking were ranked with CONSRANK40. The interface in the docking decoys were analysed, visualised and compared by the COCOMAPS41 web tools. PRISM was used for statistical analysis.

For manuscripts utilizing custom algorithms or software that are central to the research but not yet described in published literature, software must be made available to editors/reviewers. We strongly encourage code deposition in a community repository (e.g. GitHub). See the Nature Research guidelines for submitting code & software for further information.

Data

Policy information about availability of data

All manuscripts must include a data availability statement. This statement should provide the following information, where applicable:

- Accession codes, unique identifiers, or web links for publicly available datasets
- A list of figures that have associated raw data
- A description of any restrictions on data availability

The source data underlying figures 1b, d, 2a-d, 3, 4c-h, 5, Supplementary Fig. 1c, d, e, 2, 3, 4a, c, d, 6b-f, 7a, c, e, f, 8c, d, f-h, 9a, b, 10, are provided as a Source Data file.

Field-specific reporting

Please select the one below that is the best fit for your research. If you are not sure, read the appropriate sections before making your selection.

☒ Life sciences ☐ Behavioural & social sciences ☐ Ecological, evolutionary & environmental sciences

For a reference copy of the document with all sections, see nature.com/documents/nr-reporting-summary-flat.pdf

Life sciences study design

All studies must disclose on these points even when the disclosure is negative.

Sample size	Statistical methods were not used to determine sample sizes.
Data exclusions	No data were excluded.
Replication	All experiments were reliably reproduced to support the conclusions stated in the manuscript.
Randomization	Randomization was not applicable to this study.
Blinding	Investigators were not blinded.

Reporting for specific materials, systems and methods

We require information from authors about some types of materials, experimental systems and methods used in many studies. Here, indicate whether each material, system or method listed is relevant to your study. If you are not sure if a list item applies to your research, read the appropriate section before selecting a response.

Materials & experimental systems

n/a	Involved in the study	
☐	☒	Antibodies
☐	☒	Eukaryotic cell lines
☒	☐	Palaeontology
☒	☐	Animals and other organisms
☒	☐	Human research participants
☒	☐	Clinical data

Methods

n/a	Involved in the study	
☒	☐	ChIP-seq
☒	☐	Flow cytometry
☒	☐	MRI-based neuroimaging

Antibodies

Antibodies used	The antibodies used were as follows: anti-Banf1 N-terminus (SAB1409950, Sigma-Aldrich and ab88464, Abcam), anti-Banf1 C-terminus (PRS40170604, Sigma-Aldrich), PARP1 (9532, Cell Signaling Technology), PARP1 (ab191217, Abcam Figure 2e), anti-Emerin (5430, Cell Signalling Technology), anti-Flag M2 Antibody (F3165, Sigma-Aldrich), anti-PAR (ab14459, Abcam), anti-γ-Tubulin (T6557, Sigma-Aldrich), anti-H3 (4499, Cell Signaling Technology), anti-H4 (2935 Cell Signaling Technology), anti-SP1 (9389, Cell Signaling Technology), anti-EGFR (sc-03, Santa-cruz), anti-LC3B (2775, Cell Signaling Technologies), anti-phospho-p53 ser15 (2984, Cell Signaling Technology), anti-b-actin (612656, BD Biosciences). Fluorescent secondary antibodies used were: Donkey anti-Mouse 800 nm (LiCor; IRDye 800CW 926-32212), Donkey anti-Rabbit (LiCor; IRDye 680LT 926-28023) and Alexa Fluor 488 and 594 (Molecular Probes).
Validation	All antibodies used in the current study are commercially available. Antibodies were validated in the current study as follows; either using siRNA (PARP1, Banf1, EMD, Supplementary figures 4a, b, 8d and data not shown), Parp inhibitors (PAR Supplementary figure 2d), expression of Flag-tagged proteins (anti-Flag, Figure 2c). The antibodies against anti-H3, anti-H4, anti-SP1, anti-EGFR, anti-LC3B, anti-b-actin and anti-γ-Tubulin have been previously validated by suppliers and in the current study were confirmed to run at the expected size via immunoblotting and be present in the correct cellular fraction (Supplementary figures 1d and 9b).

Eukaryotic cell lines

Policy information about cell lines

Cell line source(s)	U2OS and HEK293T cells were obtained directly from ATCC. The NGPS patient cell lines and control cell line were obtained from the laboratory of Carlos Lopez-otin (Cabanillas, R. et al. Am J Med Genet A 155A, 2617-2625, doi:10.1002/ajmg.a.34249 (2011) and Puente, X. S. et al. Am J Hum Genet 88, 650-656, doi:10.1016/j.ajhg.2011.04.010 (2011).
Authentication	The U2OS and HEK293T cell lines used in the study were derived directly from the original authenticated early passage

Authentication	number freeze -downs from the ATCC. The NGPS cells were confirmed to have the A12T Banf1 mutation by the lack of antigenicity with the N-term Banf1 antibody (as observed in Puente at al) and subsequent detection of the mutant Banf1 using the C-term Banf1 antibody.
Mycoplasma contamination	All cell lines were confirmed to be mycoplasma free.
Commonly misidentified lines (See ICLAC register)	*Name any commonly misidentified cell lines used in the study and provide a rationale for their use.*
